# Projected Impact of Dengue Vaccination in Yucatán, Mexico

**DOI:** 10.1371/journal.pntd.0004661

**Published:** 2016-05-26

**Authors:** Thomas J. Hladish, Carl A. B. Pearson, Dennis L. Chao, Diana Patricia Rojas, Gabriel L. Recchia, Héctor Gómez-Dantés, M. Elizabeth Halloran, Juliet R. C. Pulliam, Ira M. Longini

**Affiliations:** 1 Department of Biology, University of Florida, Gainesville, Florida, United States of America; 2 Emerging Pathogens Institute, University of Florida, Gainesville, Florida, United States of America; 3 Vaccine and Infectious Disease Division, Fred Hutchinson Cancer Research Center, Seattle, Washington, United States of America; 4 Department of Epidemiology, University of Florida, Gainesville, Florida, United States of America; 5 Institute for Intelligent Systems, University of Memphis, Memphis, Tennessee, United States of America; 6 Health Systems Research Center, National Institute of Public Health, Cuernavaca, Morelos, Mexico; 7 Center for Inference and Dynamics of Infectious Diseases, Seattle, Washington, United States of America; 8 Department of Biostatistics, University of Washington, Seattle, Washington, United States of America; 9 Department of Biostatistics, University of Florida, Gainesville, Florida, United States of America; Oswaldo Cruz Foundation, BRAZIL

## Abstract

Dengue vaccines will soon provide a new tool for reducing dengue disease, but the effectiveness of widespread vaccination campaigns has not yet been determined. We developed an agent-based dengue model representing movement of and transmission dynamics among people and mosquitoes in Yucatán, Mexico, and simulated various vaccine scenarios to evaluate effectiveness under those conditions. This model includes detailed spatial representation of the Yucatán population, including the location and movement of 1.8 million people between 375,000 households and 100,000 workplaces and schools. Where possible, we designed the model to use data sources with international coverage, to simplify re-parameterization for other regions. The simulation and analysis integrate 35 years of mild and severe case data (including dengue serotype when available), results of a seroprevalence survey, satellite imagery, and climatological, census, and economic data. To fit model parameters that are not directly informed by available data, such as disease reporting rates and dengue transmission parameters, we developed a parameter estimation toolkit called AbcSmc, which we have made publicly available. After fitting the simulation model to dengue case data, we forecasted transmission and assessed the relative effectiveness of several vaccination strategies over a 20 year period. Vaccine efficacy is based on phase III trial results for the Sanofi-Pasteur vaccine, Dengvaxia. We consider routine vaccination of 2, 9, or 16 year-olds, with and without a one-time catch-up campaign to age 30. Because the durability of Dengvaxia is not yet established, we consider hypothetical vaccines that confer either durable or waning immunity, and we evaluate the use of booster doses to counter waning. We find that plausible vaccination scenarios with a durable vaccine reduce annual dengue incidence by as much as 80% within five years. However, if vaccine efficacy wanes after administration, we find that there can be years with larger epidemics than would occur without any vaccination, and that vaccine booster doses are necessary to prevent this outcome.

## Author Summary

Dengue is a mosquito-transmitted viral disease that is common throughout the tropics. Despite a long history in humans and extensive efforts to control dengue transmission in many countries, the number, severity, and geographic range of reported cases is increasing. Most control efforts have focused on controlling mosquito populations, but the main vector, *Aedes aegypti*, flourishes in human-disturbed and indoor environments. Because the mosquitoes prefer to bite during the day when people are active and potentially moving around high-risk locations, fixed barriers like bed nets are not effective. Several dengue vaccines are being actively developed and may become valuable tools in dengue control. Using historical dengue data from Yucatán, Mexico, we fit a detailed simulation of human and mosquito populations to project future transmission, then used efficacy data from vaccine trials to evaluate the benefit of potential vaccination deployment strategies in the region. For a durable vaccine, we find that population-level, annual vaccine effectiveness approaches 65% by the end of the 20-year forecast period. For waning vaccines, however, effectiveness is greatly reduced–and sometimes negative–unless booster vaccinations are used.

## Introduction

Dengue is currently the most important arboviral disease of humans and has an increasing global public health burden [1]. Worldwide, the combined annual number of infections by the four dengue serotypes has been estimated to be close to 400 million, of which 96 million develop symptomatic disease [2]. Globally, dengue incidence has consistently increased for the last five decades due to geographic expansion and transmission intensification in endemic tropical and subtropical regions [3–6]. Since individuals may be infected multiple times with different viral serotypes, and because re-infection is associated with an increased risk for severe disease, dengue presents unique challenges for prevention and control [7].

Vector control is the only option currently practiced to reduce dengue transmission, with most efforts targeting *Aedes aegypti* and *Ae. albopictus*, but these programs provide limited protection and may not be sustainable. Most communities undertaking vector control lack the budget, personnel, and expertise needed to effectively reduce mosquito populations. Although the use of DDT as a vector control measure substantially reduced dengue transmission in the 1960’s and 70’s, vector control efforts in the post-DDT era have not been sufficient to prevent invasion of dengue into new regions [8–10].

Vaccination may soon be available as an additional option for dengue intervention. Six vaccines are in clinical development, but to date only the Sanofi-Pasteur vaccine, Dengvaxia, has completed phase III trials [11]. Phase III trials conducted in Latin America estimated vaccine efficacy of 64.7% (95%CI [58.7, 69.8]), while the estimate from a trial in South East Asia was 56.5% (95%CI [43.8, 66.4]). Pooled analysis of these two trials indicates vaccine efficacy is significantly higher for participants with pre-existing dengue neutralizing antibodies (81.9%; 95%CI [67.2, 90.0]) compared to those who were seronegative at the time of vaccination (52.5%; 95%CI [5.9, 76.1]). Vaccine efficacy against hospitalization for dengue in Latin America was 80.3% (95%CI [64.7, 89.5]) and in South East Asia was 67.2% (95%CI [50.3, 78.6]), and vaccine efficacy estimates varied by serotype in both trials [12–14].

Overall, these are promising results for Dengvaxia, but trial outcomes have been mixed. Efficacy appears to decline in recipients that have not had a previous natural infection, including an apparent increased risk of hospitalization in pre-school age children. Vaccine rollout plans will need to be carefully evaluated, particularly the age targeted for routine vaccination relative to the age by which people in the target region have typically had an infection [15–18].

To accurately model the impact of potential vaccination strategies, we must account for the primary mechanisms that influence dengue transmission: mosquito population dynamics and behavior [10, 19, 20], seasonal influences on these factors [21, 22], human movement and demography [23, 24], the build-up of strain-specific immunity in the population through time, and the immune response following re-exposure [6, 15, 19, 25].

Consistent with the global trend, dengue incidence and severity have increased significantly in Mexico over the past four decades, with transmission regularly reported in 28 of the 32 Mexican states. Incidence is particularly well-documented in the state of Yucatán. Dengue was reintroduced to Yucatán in 1979 after widespread DDT use in the region ceased; the virus had not been detected in the state for the previous two decades [26, 27].

We use an agent-based simulation model fitted to data on dengue occurrence to examine the possible effectiveness of deploying a Dengvaxia-like vaccine in Yucatán. We compare potential vaccine rollout strategies under varying assumptions regarding the duration of vaccine-induced immunity. In particular, we consider routine vaccination targeting different age groups (2, 9, or 16 year olds), with and without one-time catch-up campaigns, and with durable or waning vaccine-induced immunity. Our transmission model extends previously published work that examined dengue vaccination in Thailand with a hypothetical vaccine [28].

## Methods

We consider vaccine interventions using a stochastic, discrete-time, agent-based model of dengue transmission that explicitly represents humans, infected mosquitoes, and transmission of dengue virus serotypes between them. The overview below summarizes the current version of the model and describes important changes made to the previously published version of the model, which was developed to study dengue in Thailand [28].

We compile the model using GCC [29], importing GSL [30] for pseudo-random number generation, and perform simulations on the University of Florida High Performance Computing Cluster. On an AMD Opteron 4284, each model run takes ∼2 minutes per simulated year for the Yucatán population (1.82 million); run time is approximately linear with population size for a given set of transmission parameters. Memory use is also linear with population, and is ∼0.7 GB for Yucatán. The C++ source code is available at https://github.com/tjhladish/dengue/releases/tag/v2.0. We use R for analysis of simulated data and figure generation [31].

We fit the model to long-term clinical and severe case data (Table B in S1 Text) [32] and a 1987 serosurvey [33, 34] using AbcSmc [35], a C++ implementation of Approximate Bayesian Computation Sequential Monte Carlo [36].

Our model, described below, reflects current understanding of dengue in the region, specifically dengue natural history, the local human and mosquito populations, and a model of the vaccine which reflects general results for Dengvaxia, but is not intended to explain all of the trial outcomes. In general, the model features and parameters should not be interpreted literally (*e.g.* the number of mosquitoes in the model is unlikely to be the actual number of mosquitoes in the Yucatán). Rather, when taken as a whole, they are effective values related to dengue transmission given our other assumptions. They should not be used in other modeling work without considering this context.

### Dengue Natural History Model

**Fig 1:**
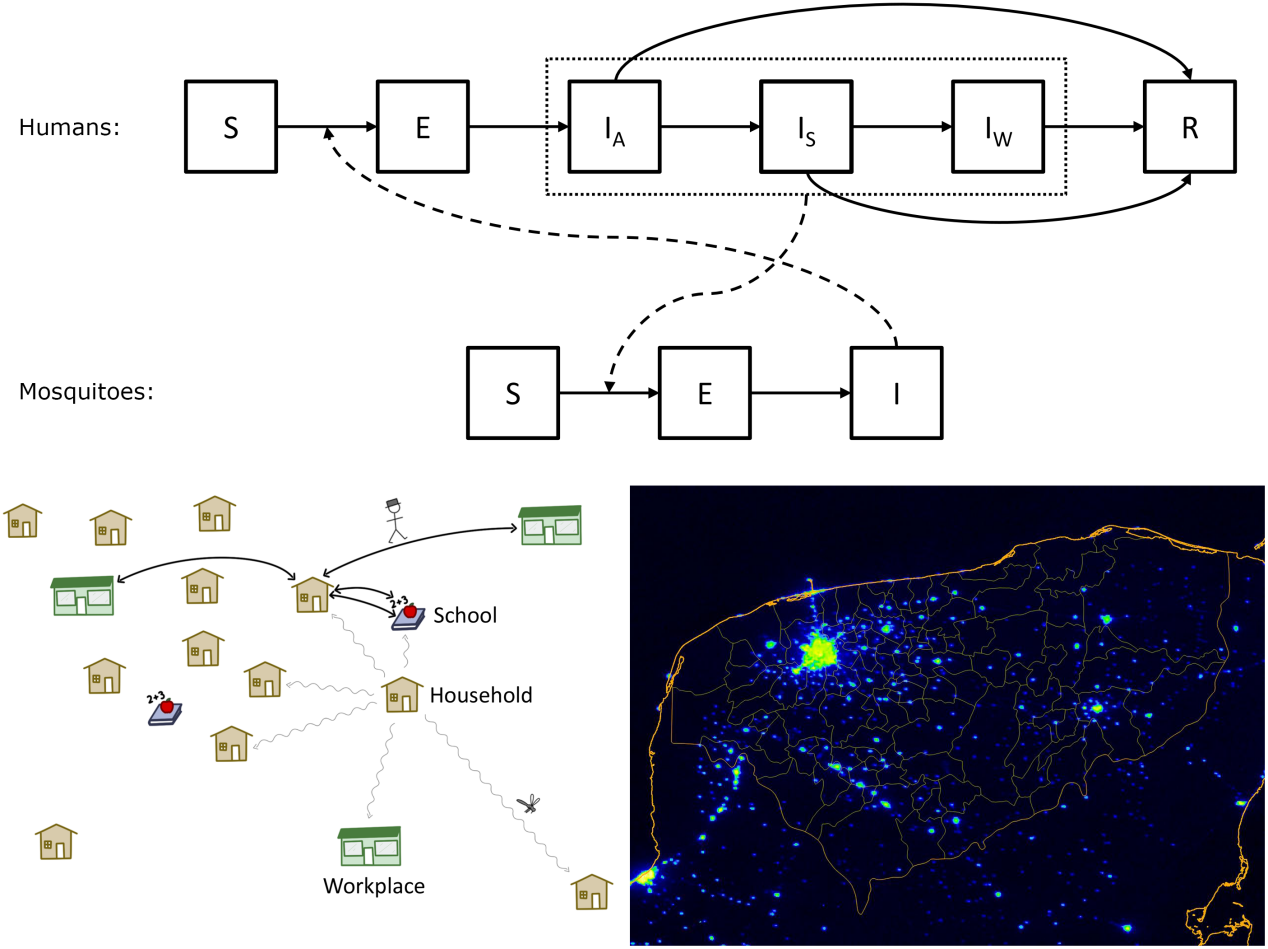
Epidemic and synthetic population model structures. Top: Boxes represent disease states that individual humans (**S**usceptible, **E**xposed, **I**nfectious [**A**symptomatic, **S**ymptomatic, **W**ithdrawn], and **R**esistant) and mosquitoes (**S**usceptible, **E**xposed, **I**nfectious) may progress through. Solid arrows denote possible transitions between states. Dashed arrows denote transmission of the virus between mosquitoes and humans. Bottom-left: A cartoon representation of the synthetic population. People move daily to work or school (smooth arrows), and mosquitoes move randomly between adjacent locations (wavy arrows). Bottom-right: False color nighttime-light satellite image of Yucatán, Mexico and surrounding regions, an indicator of human population density. Nighttime light data is used to determine the placement of households in the model. The orange lines denote coastline (thickest), state of Yucatán (middle), and municipal (thinnest) boundaries. Yellow indicates highest population density, dark blue indicates lowest. The bright area in the upper-left quadrant is the state capital city, Mérida.

Uninfected mosquitoes acquire dengue virus by biting infectious humans. Infected mosquitoes that survive the extrinsic incubation period (EIP) can infect new hosts on subsequent blood-feeding attempts (Fig 1; see Section S1 in S1 Text). Infected humans also incubate the pathogen before they become infectious. Dengue virus comprises four serologically distinguishable lineages, called serotypes. Infection produces lifelong immunity to the infecting serotype and induces temporary cross-protection against other serotypes, but can later enhance severity in a subsequent infection, an effect referred to as antibody-dependent enhancement (ADE). ADE also occurs in infants due to maternal antibodies [37]. We represent infection with all four serotypes, with different disease outcome probabilities depending on number of previous infections. Different disease outcomes (asymptomatic, mild, and severe) also influence transmission, since they have shorter (asymptomatic) to longer (severe) infectious periods.

In our model, seasonal rain reliability determines mosquito population size and temperature determines EIP. With these seasonality drivers, after fitting we find the dengue basic reproduction number (*R*_0_) falls below 1 for roughly four months each year, indicating that trans-seasonal maintenance via transmission in the local human population is unlikely. The mechanism that causes dengue to re-emerge after the winter in Yucatán is unknown. Plausible explanations include but are not limited to human movements from regions with on-going transmission, infected mosquitoes introduced via *e.g.* freight, and vertical transmission in the vector. We address seasonal re-emergence using a small, fixed daily exposure probability to represent whatever the real processes are. The serotypes for these exposures are based on the observed serotypes in Yucatán.

We classify infections as asymptomatic (“inapparent”) or symptomatic (a “case”). Cases are further separated into mild or severe, and for comparisons with empirical data, we assume that mild and severe cases can have different reporting rates. We model the pathogenicity (probability an infection is symptomatic) as a reference probability combined with relative-risk factors for particular serotypes, past infection history, and age. We use a similar approach for the probability of severe disease. Finally, in our model infants (people <1 year old) may have maternal antibodies, which causes them to either resist infection or have enhanced pathogenicity (see Section S1 in S1 Text).

### Synthetic Human Population

We explicitly model individuals and their activity in a synthetic population within Yucatán, based on census and household data [38, 39], and national statistics on local economies and schools [40, 41]. Individuals have a fixed age, gender, and household, and may travel to school or work during the day. We use gender to determine a mother-child relationship when an infant is exposed so that we can consider maternal antibodies, because the model population structure specifies cohabitation but not familial relationship.

For practical reasons, we do not consider changing population size or age structure: fixing these across all simulations dramatically reduces the model’s computational complexity. Household composition and location, as well as associated schools or workplaces, are also static and identical across all simulations. These locations and the distances between them provide all the spatial information in the model, as humans and mosquitoes strictly exist at and move between these places. Using such locations for spatial distribution is convenient because household, school, and workplace data are available from national and international sources, and because it is a natural approximation of where people and mosquitoes interact.

Representing how acquired immunity changes over time in a population is critical for modeling long-term epidemic dynamics. To avoid the complexity of a dynamic demographic model, instead of having individuals age, immune histories are annually shifted from younger to older individuals. Thus, the population matures by accumulating immunity as epidemics occur, while maintaining a realistic household age distribution. Because the size of age cohorts trends downward with increasing age, mature immune histories are implicitly lost due to mortality and replaced with newborns who have no acquired immunity, though they may have temporary maternally-derived immune responses (see Section S2 in S1 Text).

### Synthetic Mosquito Population

**Fig 2:**
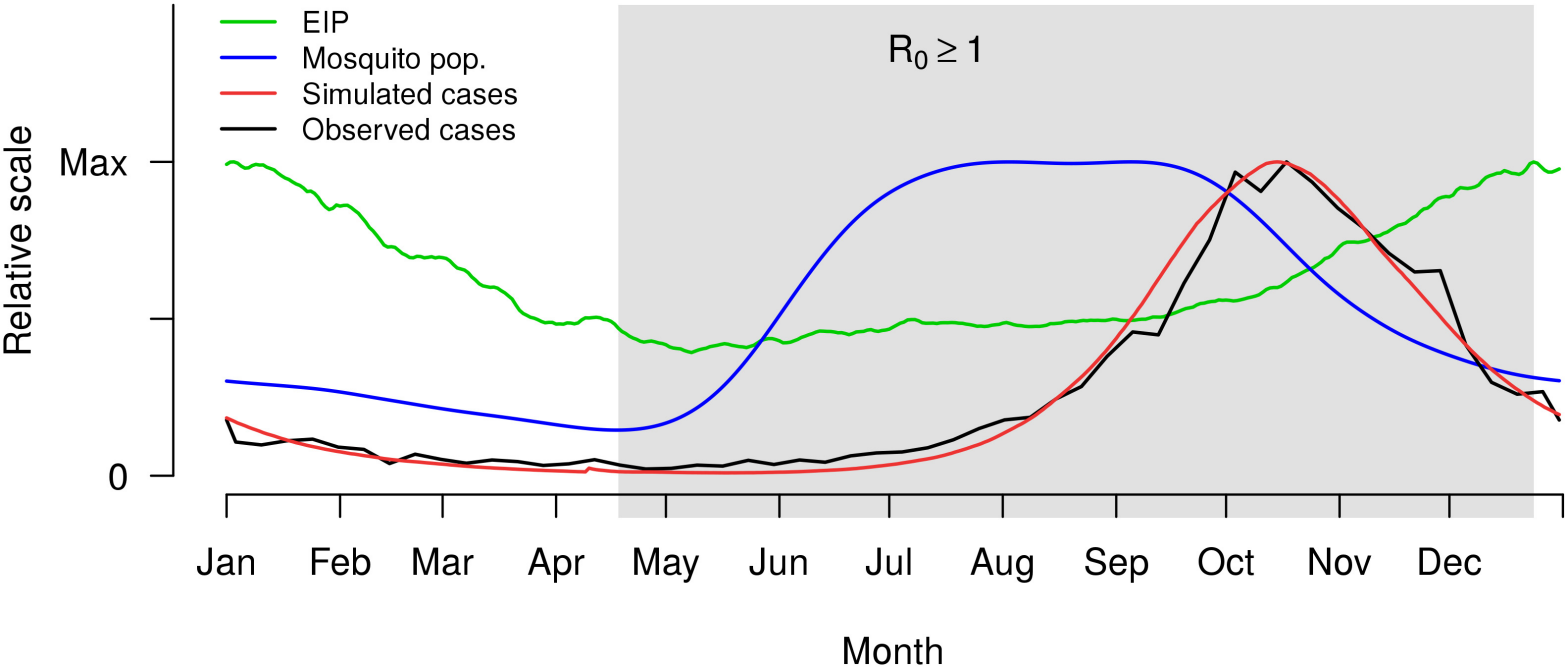
Seasonal changes in EIP (driven by temperature data) and mosquito population size (driven by precipitation data), combined with immunological dynamics within the human population, produce seasonality of simulated dengue cases that matches the seasonal pattern of reported case data for Yucatán, Mexico. This annual pattern is not part of the fitting procedure, but is successfully replicated by the model. Curves are normalized by their maximum values to highlight the seasonal relationships between datasets. The shaded region indicates the time of year when the basic reproduction number, *R*_0_, exceeds 1. The simulated and observed cases are averaged for the available weekly data (1995–2011 only). Observed cases are weekly aggregates; all other data are daily.

We model the mosquito population in two parts: aggregate populations for uninfected (susceptible) mosquitoes at each location, and mobile agents for infected mosquitoes. The aggregate mosquito populations have location-specific sizes drawn from an exponential distribution with a fitted mean (see Section S5 in S1 Text) that varies seasonally as a function of rainfall (Fig 2).

Upon infection, individual mosquitoes are separated from the aggregate population. The mosquito’s age is determined by sampling from the total mosquito age distribution. Her EIP is then drawn from the day’s EIP distribution (based on effective temperature) and added to her age to determine at what age she will become infectious. Finally, the mosquito’s age-at-death is determined by sampling from the distribution of mosquito ages greater than or equal to the current age (see Section S3 in S1 Text). Mosquitoes that will die before becoming infectious cannot contribute to disease transmission and effectively die instantaneously. Individual mosquitoes may move between houses, workplaces and schools; if they do move, they randomly select from adjacent locations weighted by the inverse distance-squared distance to those locations.

### Time

Simulation time has important features at several scales: daily day-night cycles, seasonal change in temperature and rainfall by day-of-year, annual population turnover, and multi-year eras.

Each day bridges a day-night cycle, 7 a.m.–7 a.m. People go to work or school (or stay home if not employed) during the day and are at their homes during the night; there is no representation of weekends or holidays. Individuals’ daily movement patterns sometimes change in response to disease (see Section S2 in S1 Text), but not otherwise. Mosquitoes are more likely to bite during the day than at night.

Seasonal effects on the mosquito population (driven by precipitation patterns) and on the EIP (driven by temperature) change daily, based on a time series generated from 35 years of historical temperature [42] and precipitation [43] data.

Each year, the human population ages on the same day of the year (Julian day 99; *i.e.*, April 9), which is roughly the nadir in transmission. Leap-days are not modeled: each year is 365 days long. We do not adjust the observed data to match this assumption.

At the multi-year scale, there are four time periods: (1) a priming period, to establish a stable distribution of acquired immunity in the population; (2) an intense vector control period, corresponding to the use of DDT in Yucatán (1956 to 1978); (3) a fitting period (1979 [first recorded Yucatán dengue epidemic] to 2013 [last year with complete data]); and finally (4) a 20 year forecast period (2014 to 2033) where we consider vaccination strategies. All periods (priming, DDT era, fitting, and forecast) are simulated with the same parameters in a particular run, except that during the DDT period, both mosquito populations and external introductions are reduced. We represent vector control by reducing the mosquito populations by 77% and the rate of dengue introductions by 90%, based on the fraction of households that were treated with DDT and other insecticides in Yucatán and neighboring states, respectively, from 1956 to 1978 [44].

### Vaccination

In our model, vaccination reduces, but does not eliminate, the probability of infection with dengue given exposure, and has no other direct effect on transmission or disease. Vaccination status is added to an individual’s immune history but is distinct from immunity acquired by natural infection. We consider vaccines that confer durable (lifelong) protection, as well as the possibility that vaccine-induced protection declines linearly (“wanes”) with the number of days since vaccination. We consider three possible waning half-lives.

We assume vaccine efficacy (*VE*) values consistent with the the phase III trial results for Dengvaxia in Latin America [12, 13] (see Section S4 in S1 Text). Trial results indicated that prior dengue infection approximately doubles the vaccine efficacy. Table 1 gives the values used in the model.

**Table 1:** Assumed Vaccine Efficacy for Susceptibility (*VE*_*S*_) for naïve and previously infected individuals, estimated from the overall efficacy found by the Sanofi-Pasteur phase III vaccine trial. Efficacies for naïve and previously infected individuals are calculated assuming a population that is 60% antibody positive and that efficacy in seropositive individuals is twice that of seronegatives.

	DENV1	DENV2	DENV3	DENV4
Overall (observed)	0.5	0.42	0.74	0.78
Fully naïve (imputed)	0.3	0.27	0.45	0.48
Previous infection (imputed)	0.6	0.54	0.9	0.95

Dengvaxia has a three dose schedule, each six months apart. We also model a 3 dose regimen, with the first dose providing full efficacy, and all vaccinees receiving all three doses. For scenarios where the vaccine wanes, it does so between doses as well, but each dose is assumed to return efficacy back to the initial level.

During the forecast period (2014–2033), individuals in an age category (2, 9, or 16 year-olds) are targeted for routine vaccination annually on Julian day 100, one day after immune histories are transferred. We also consider scenarios in which routine vaccination is supplemented with a one-time catch-up campaign, where vaccination occurs across many age groups (from one year older than the routine age, up to age 30) in the first year of the forecast period. When considering booster strategies for a waning vaccine, booster doses are provided to all previously vaccinated individuals, every two years (irrespective of waning period) from the final dose date.

Because the sizes of our age groups differ, we cannot simply hold the fraction of individuals vaccinated in an age group (*i.e.*, coverage) constant while assessing the outcomes for targeting different ages: at the population level, this would be assessing different vaccination rates and confound with other differences due to age. Instead, we hold the number of doses constant across routine strategies, and across catch-up campaigns when used. However, we base the number of doses on attaining 80% coverage in 9 year olds when targeting that age (30,100 vaccinees), and 60% coverage in the catch-up cohort associated with 9 year olds (448,500 vaccinees).

While we have represented some of the features of the Sanofi-Pasteur vaccine, our intent is to represent a generic, moderate-efficacy vaccine. We assume that vaccine performance is affected by serostatus but not age of vaccinee *per se*, as that has not been specifically tested in the trials. We do not address the potential complexities indicated by trial results in Southeast Asia, particularly any potential for disease enhancement [14].

### Effectiveness

We assess vaccination strategies by contrasting the projected dengue burden with and without vaccine deployment over a 20 year forecast period. Matched baseline and vaccination scenario runs are simulated for the forecast period, with these comparison runs sharing parameters produced by the fitting procedure (see Section S5 in S1 Text) as well as simulated history for the priming, vector control, and fitting periods. We average across 1000 runs (100 parameter combinations times 10 samples each) of the baseline and each scenario to get an expected number of cases each year.

In a time interval Δ*t*, the total vaccine effectiveness (*V*_eff,Δ*t*_) is 1 minus the proportion of the symptomatic cases in the vaccination scenario (*VS*_*Δt*_) relative to the number in a baseline with no intervention (*B*_Δ*t*_):

$$V_{\text{eff},\Delta t}=1-\frac{{\mathrm{VS}}_{\Delta t}}{B_{\Delta t}}$$ (1)

We calculate this value for an annual (Δ*t* = 1 year, beginning 0, 1, 2, …, 19 years after initiation of the intervention) and cumulative (Δ*t* = 1, 2, …, 20 years, all from initiation of the intervention) basis, across parameter combinations and replicates.

### Parameterization

The model uses parameters from a wide range of sources (see Section S5 in S1 Text). Our synthetic human population was constructed using satellite imagery, microcensus, workplace and school data (see Synthetic Human Population). Seasonality in mosquito population and EIP is based on empirical temperature and precipitation time series (see Section S3 in S1 Text). When data were not available to inform or fit the model directly, as with certain vaccine performance parameters, we made assumptions that simplified the model implementation.

**Fig 3:**
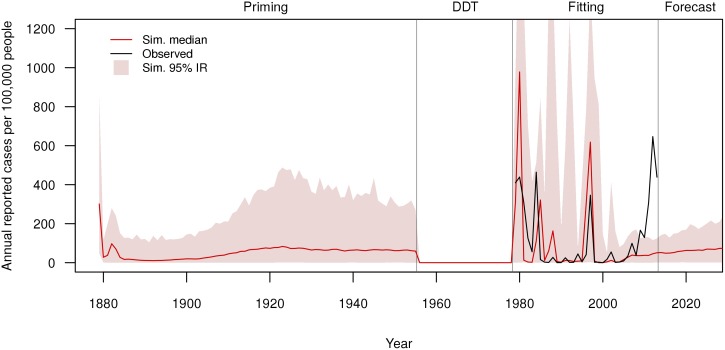
Observed (black line) versus simulated median (red), and 95% interquantile range (IR, pink region) of reported (per 100,000 people) annual dengue incidence in the Yucatán population. Population immunity for the fitting era is established by running the model for 100 years (“priming” and “DDT” eras). Empirical case and serotype data are not available prior to 1979 for Yucatán. The model is fit to per capita incidence (1979–2013), and we consider possible interventions during a 20-year projection period (2014–2033; baseline without intervention shown).

Epidemiological, entomological, and vaccine parameters were taken from the literature, or fit using Approximate Bayesian Computation (see Table D in S1 Text). We fit our model to reported case and seroprevalence data collected between 1979 and 2013 (the fitting period, main text Fig 3 and Fig. P in S1 Text). We retained the 100 best-performing parameter combinations (out of 70,000–10,000 per set, 7 sets) from the fitting procedure. In our forecasts, we used each of those 100 parameter combinations 10 times, for a total of 1000 replicates.

### *R*_0_ Estimation

Since transmission parameters vary seasonally, we estimate the basic reproduction number, *R*_0_, by day of year. For a vector-borne disease, *R*_0_ may be thought of as the number of additional human infections that are expected to result from a single infected human in a naïve population, after exactly one human-mosquito-human transmission cycle. *R*_0_ does not take into account existing immunity in a population, but it nonetheless can provide some intuition about the seasonal timing and peak size of an epidemic. We estimate *R*_0_ as follows: for each day of the year, we randomly infect an individual in an otherwise completely susceptible population, allow that person but no other people to infect mosquitoes, run the simulation forward until all infections clear, and count all human infections after the first. We do this for the same 1000 samples used in the forecasting and average the number of secondary infections across the samples to compute *R*_0_ for that day.

## Results

### Baseline Dynamics

Using the best parameter combinations from our fitting procedure, we simulated the historical and fitted period outbreaks to establish background immune profiles. Our model generally predicts the size and timing of epidemics during most of the fitting period (1979–2008), but not the large epidemics since 2009. We also forecast transmission from 2014 through 2033 (the 20 year forecast period) without any intervention to provide a baseline to compare interventions against. Median results are reported here, and prediction intervals can be found in the supplement (see Section S7 in S1 Text).

To generally characterize dengue transmission in the region, we calculate the seasonally-varying *R*_0_ for DENV1. We estimated a seasonal peak *R*_0_ of 5.2, occurring in August (Fig. A in S1 Text, panel C). From late December through mid April, *R*_0_ is below 1.0. Dengue introductions to the population in the model can happen throughout the year, so stuttering transmission chains are still observed in those months. We did not repeat this analysis for all serotypes, but the others would have generally lower *R*_0_ given our assumption that DENV1 has the highest risk of severe disease, and thus longer infectious periods than the other serotypes.

We evaluate the performance of the fitted model using seasonality and seroprevalence data that were not used in the fitting procedure. Precipitation and temperature seasonality in the model (see Section S3 in S1 Text) drives changing mosquito populations and changing EIPs (Fig. A in S1 Text). These seasonal effects successfully reproduce the overall shape and timing of average weekly dengue incidence (Fig 2, average of years for which weekly data are available).

**Fig 4:**
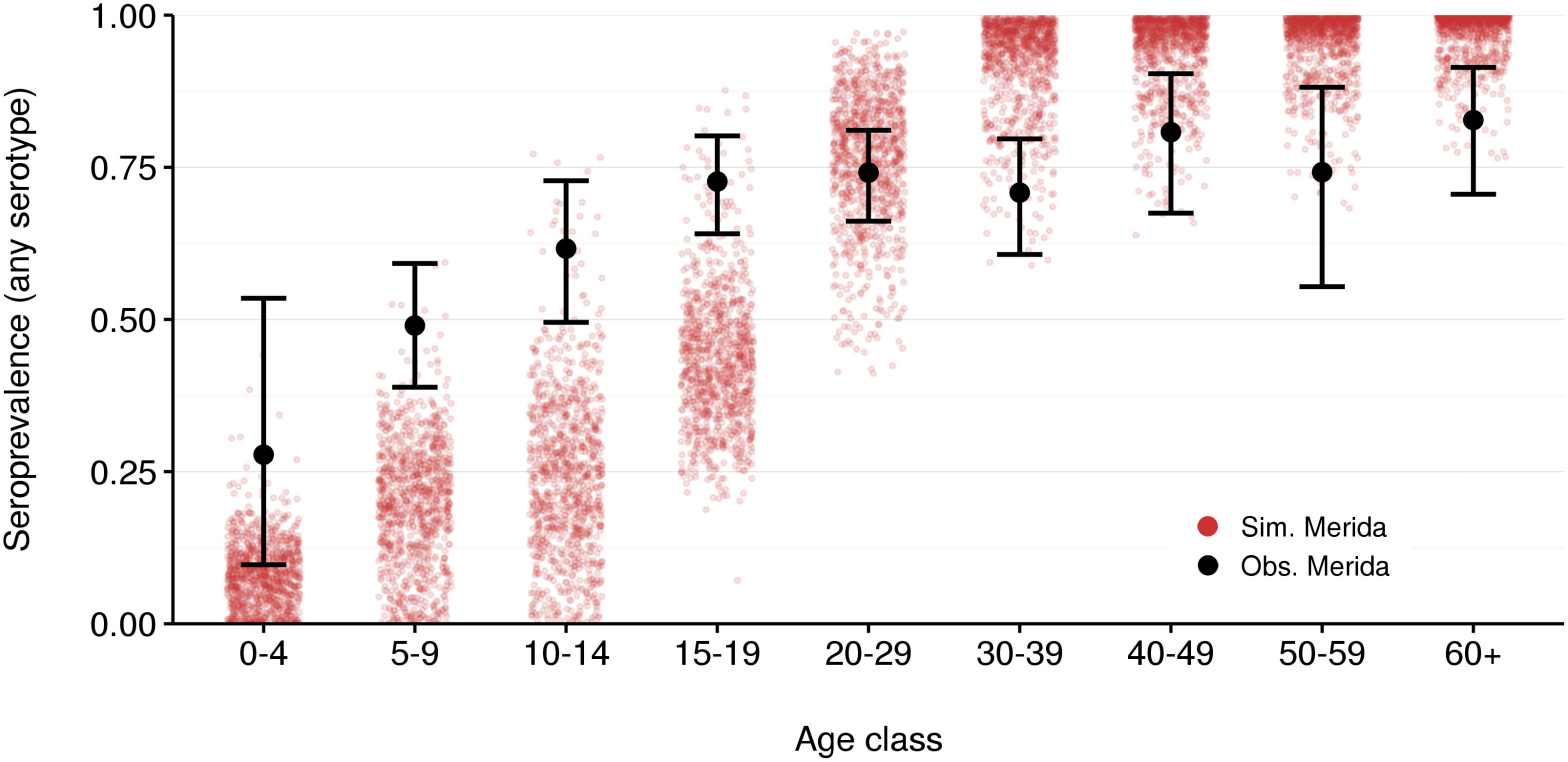
Seroprevalence by age group, observed [45, 46] and simulated. Seroprevalence (seropositive fraction, any serotype) from Mérida, Yucatán was recorded in 2014 (686 total participants). Red dots indicate simulated population seroprevalence on April 10, 2014. Error bars indicate 95% confidence intervals. This comparison is for qualitative validation only: these data are not used in fitting. Our model results are low for potential vaccinees; however, this should provide a conservative estimate of vaccine effectiveness, since the model parameterization assumes efficacy is higher in seropositive individuals.

Seroprevalence, or the fraction of individuals with at least one past dengue infection, is a general indicator of whether an epidemic is possible and how large one might be if it occurs. This relationship is more complicated for dengue given the temporary nature of cross-protection between serotypes and subsequent disease-enhancement, but still provides some insight. To qualitatively assess our model fit, we compared age-stratified results from a recent serosurvey of Mérida [45, 46], the largest city in Yucatán, with simulated seroprevalence among Mérida residents in the synthetic population (Fig 4). Our model results overlap with confidence intervals for the measurements, but are generally low for individuals below age 20 and high for those over 30.

### Effectiveness of Vaccination Strategies

**Fig 5:**
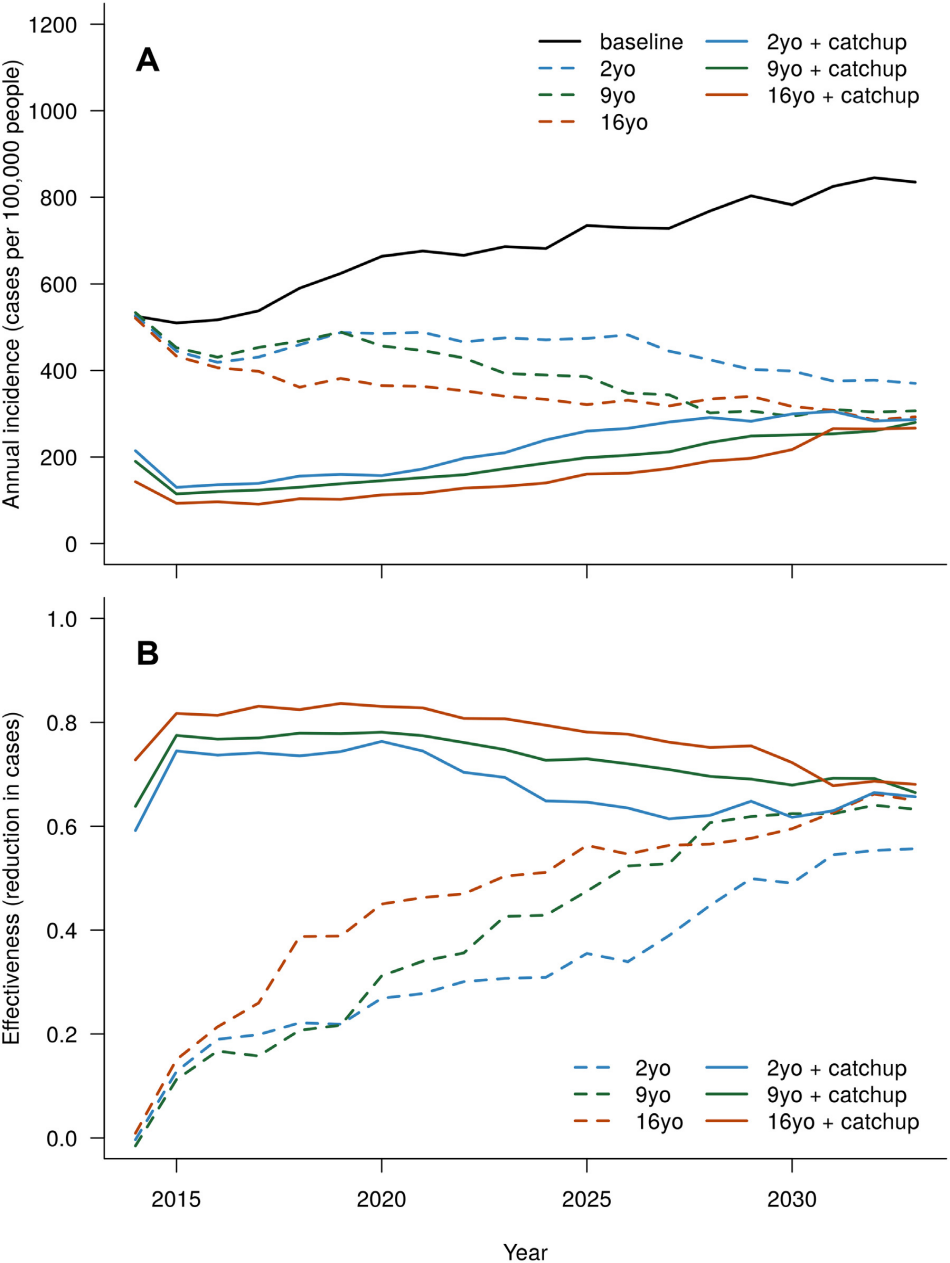
Simulated effect of a durable vaccine on total cases: (A) annual incidence and (B) effectiveness, or fraction of cases averted relative to the baseline scenario (*i.e.* no vaccination). 2, 9, and 16yo indicate the age in years of the group that is routinely vaccinated. Catch-up indicates a one time mass-vaccination campaign of individuals aged between one year over the target age for routine vaccination, and 30 years old, inclusive. Coverage is 80% for routine 9-year-old vaccination and 60% for 10- to 30-year-old catch-up. Coverage is adjusted so that all routine-only scenarios use the same expected number of vaccinations, and all scenarios with catch-up use the same expected number of vaccinations (see Section S4 in S1 Text).

For scenarios that assumed vaccine-induced immunity did not wane over time (a durable vaccine), annual effectiveness gradually increased for routine-only strategies (Fig 5) and spiked early with catch-up campaigns followed by short increasing trend, then a gradual decline to roughly the same level as achieved with routine-only vaccination. Routine-only vaccination started near 0% and increased to 65% annual effectiveness, while strategies with catch-up started near 65% and quickly increased to 75%, but after about 7 years, began to decrease to 65% by end of the forecast period.

Varying the target age for routine vaccination had a modest impact on annual effectiveness (Table 2), although strategies targeting older children generally out-performed those targeting younger children. We expect a positive correlation between target age and effectiveness, based on the anticipated trend in seroprevalence with age (confirmed for this population at the outset of the forecast period; see Fig 4) and our assumption that antibody-primed vaccinees benefit from enhanced vaccine efficacy. However, annual effectiveness for all strategies appeared to be converging by the end of the forecast period.

**Table 2:** Median cumulative effectiveness, or fraction of all symptomatic (reported and unreported) cases averted over the 20 year forecast period (see Section S7 in S1 Text). The median cumulative simulated number of cases in the baseline was 286,000 in the population of 1.82 million people, or 786 per 100,000 per year. After taking into account estimated reporting rates for mild and severe cases, we forecast 75 reported cases per 100,000 per year.

Routine vaccination age	2	9	16
Routine only	0.35	0.42	0.47
Routine with catch-up	0.65	0.70	0.74

We also considered vaccine-induced immunity that wanes linearly over time (Fig. D in S1 Text), with three different half lives. Under the 2 year half-life waning model, for example, vaccinees have immunity based on Table 1 immediately after each dose, and that efficacy declines linearly each day until it is 0 at 4 years post-vaccination.

**Fig 6:**
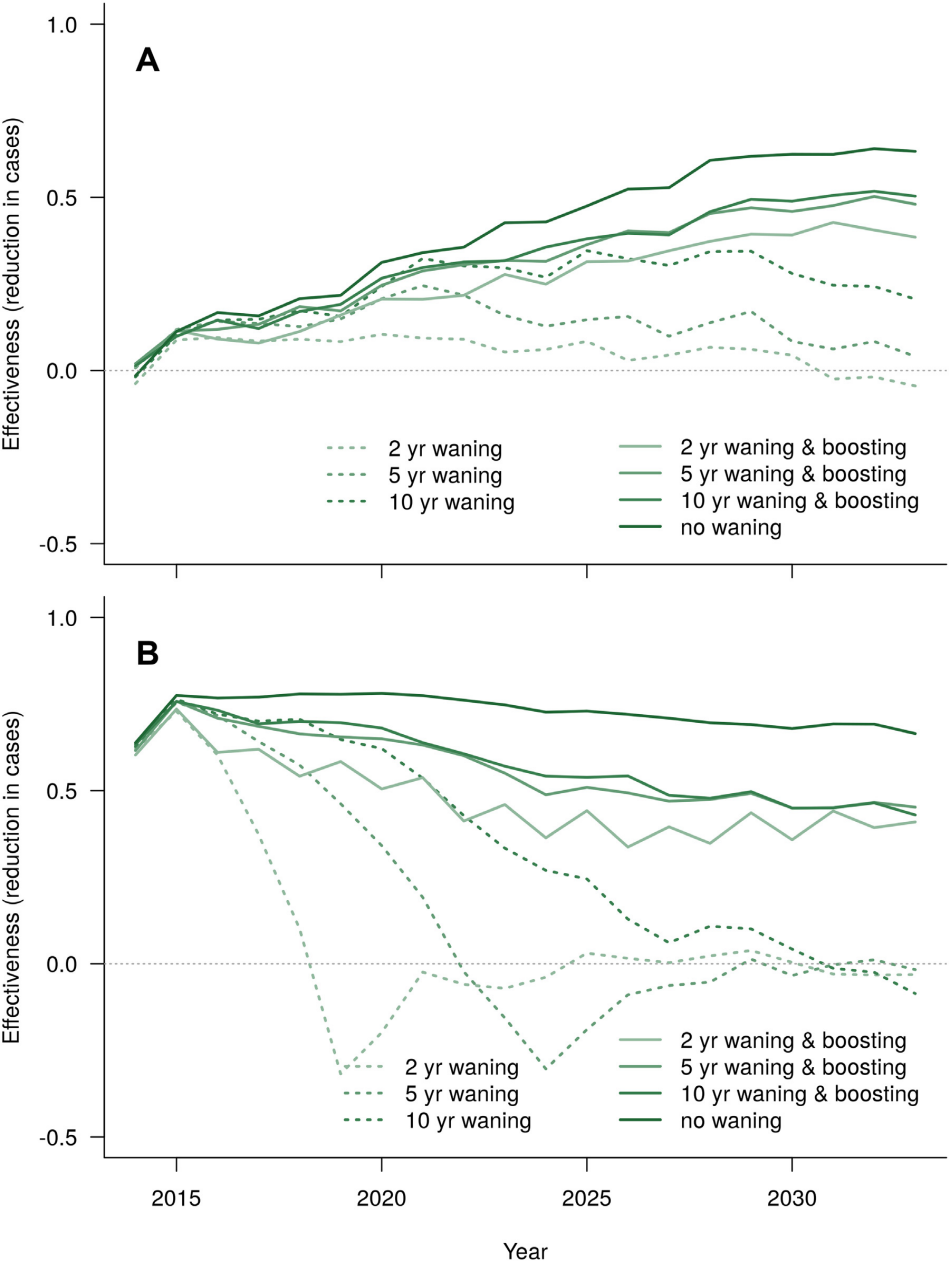
Effectiveness given waning vaccine-induced immunity for (A) routine-only vaccination of 9 year olds and (B) routine + catch-up. Administering booster vaccines at 2-year intervals substantially improves performance (three lighter solid lines), but not to the level observed with a perfectly durable vaccine (darkest solid line).

Waning substantially reduced the long-term effectiveness of routine strategies that did not include additional booster vaccinations (dashed lines, Fig 6A). When vaccinees were re-vaccinated every 2 years, performance improved, reaching 50% effectiveness after 20 years. Waning had a more dramatic effect on strategies with catch-up (Fig 6B). Catch-up campaigns with waning vaccines but no booster vaccination all resulted in negative annual effectiveness—performance worse than baseline—at some point within 20 years. That outcome is the effect of delaying cases in the catch-up cohort: annual effectiveness initially looks good as cases are temporarily prevented relative to the baseline, but when the vaccine effect fades, vaccinees that have avoided natural immunizing infections soon experience infections that have already happened in the baseline, resulting in excess cases in later years. However, cumulative effectiveness shows net case reduction–*i.e.* some of the cases are actually eliminated rather than just delayed (see Section S7 in S1 Text). Booster vaccination prevented negative annual effectiveness (lighter solid lines), but overall performance was worse than the catch-up scenario with a durable vaccine (dark solid line). The rate at which immunity wanes was an important factor for strategies without booster vaccination, but not for those with it.

## Discussion

We fit an agent-based dengue transmission model to empirical data from Yucatán, Mexico, and then used this model to evaluate a range of vaccination scenarios. For our effectiveness analysis, we used efficacy values based on phase III clinical trial results for Dengvaxia [11–13]. We concluded that a Dengvaxia-like vaccine can be an effective tool for reducing the dengue burden, although a vaccine with waning efficacy would require a booster program. We estimated a cumulative reduction in cases of 74% over 20 years for the most favorable scenario (Table 2), and scenarios with a durable vaccine converged near 65% reduction in annual case burden after 20 years (Fig 5). Scenarios with waning vaccines required booster vaccination programs to maintain appreciable effectiveness; however, with boosting, they converge at around 50% annual effectiveness by the end of the forecast (Fig 6).

In general, vaccination strategies that include only routine vaccination at a particular age are much less effective in the first 10 years than those with a one-time catch-up (Fig 5). However, as the initial catch-up cohort shrinks as a share of all vaccinees (due to mortality and on-going routine vaccination), we expect the annual effectiveness of routine-only and catch-up strategies to converge. For a durable vaccine, our model forecasts that effectiveness will converge around 65% after roughly 20 years.

The results for a waning vaccine are more complicated, particularly when there is a catch-up campaign. To address concerns raised in a recently published analysis of long-term follow-up data from the phase III trials [14], we considered vaccines that provide protective immunity that wanes with a half-life of 2, 5, or 10 years. In that study, researchers found no significant reduction in dengue hospitalization risk for vaccinated versus control groups during the third year post-vaccination, suggesting that vaccine-induced protective immunity may begin to wane. Without other adjustments to deployment strategies, we found that vaccination in these waning scenarios provides minimal long-term benefit. Furthermore, if there was an initial catch-up campaign, some years have increased incidence relative to no vaccination, though there are still small cumulative benefits (see Section S7 in S1 Text). For these scenarios, the vaccine initially prevents large epidemics, leading to a decline in naturally acquired immunity compared to baseline scenarios. When the relatively large cohort of catch-up vaccinees then collectively loses its vaccine-induced immunity over a short period of time, larger-than-baseline epidemics can result, which leads to years with expected negative annual effectiveness. However, adding booster doses to the vaccination strategy can substantially offset waning, and results in annual effectiveness around 50% at the end of the forecast period. As expected, vaccines with longer half-lives produce better effectiveness, but all waning vaccines had low long-term effectiveness without a booster program.

As a supplementary analysis, we also considered the effect of projected temperature increase associated with climate change on these results (see Section S6 in S1 Text). This sensitivity study suggests that increasing temperature would increase the projected dengue burden, but that estimates of annual vaccination effectiveness are robust to the increasing force of infection. For other public health considerations, such as adherence to dose schedules and compliance with booster campaigns, we anticipate that changing these factors would have obvious directional effects (*e.g.* lower coverage will lead to lower effectiveness), but there are not sufficient data at this time to make meaningful quantitative predictions. While compliance rates to inform such analyses might reasonably be inferred from other vaccine programs, the most critical issue is unclear: the appropriate model of vaccine action. Until there are appropriate data on Dengvaxia performance, attempts to quantify the nuanced effects of vaccine delivery are premature.

For all of our scenarios, we assumed that the vaccine efficacy for individuals who have not had a natural infection (*i.e.*, antibody-naïve) is half of that for those who have had one (*i.e.*, antibody-primed; see Table 1). Thus, as the vaccine drives down natural infection rates, it will become less effective, lowering the long-term benefit. Previous analyses have suggested that interventions (either vector control [47] or vaccination [48]) in a population that historically experienced high force of infection would initially look effective, but then have declining benefit. We observed this effect for the scenarios with a catch-up cohort: the initially high effectiveness declines after about 10 years to what appears to be a new steady state that reflects both routine vaccination coverage and a reduced level of natural infections associated with reduced transmission (Fig 5B, solid lines).

In addition to long-term outcomes, relative vaccine efficacy also influences the effectiveness of vaccination strategies based on the target age for routine vaccination: older vaccinees are more likely to be antibody-primed. This results in higher effectiveness for these strategies, but the effect is modest in the modeled Yucatán population. Therefore, other considerations such as distribution logistics might reasonably take precedence when choosing which age group to vaccinate.

In general, our fitting procedure reproduces several features of the observed data well (Table F in S1 Text), but the model is not fit to and is not intended to replicate the exact historical time series. Dengue epidemics in Yucatán are highly variable, undoubtedly influenced by factors we do not consider (*e.g.* the circulating serotypes in adjacent regions, inter-annual environmental variation). We predict the approximate timing of peaks in 1980, 1984, and 1997 due to the introduction of serotypes that had not recently circulated, but we do not predict the large epidemics observed near the end of the fitting period (Fig 3). As a consequence, we under-predict seroprevalence in young people (Fig 4), and thus may be under-predicting short-term performance of the vaccine.

These recent large epidemics are unlikely to be driven by gradual trends, such as might be captured by improved data on natural history of dengue generally, mosquito ecology in the region, and demographic and economic trends in the region. Large epidemics after quiet years are historically associated with the introduction of novel serotypes. Thus, a more substantial change to the model, such as introducing a novel strain of DENV2 capable of re-infecting people with past DENV2 infections as suggested in [49], may be necessary to replicate the end of the fitted period and the age distribution of seroprevalence.

Despite the model’s inability to reproduce the most recent large epidemics, we believe it is informative for forecasting vaccine performance for two reasons. First, even though the vaccine has twice the efficacy for seropositive versus seronegative recipients, average efficacy is relatively insensitive to changes in seroprevalence: *e.g.* if 50% of vaccinees are seropositive, overall efficacy to DENV1 is 0.45, while reducing seroprevalence by half to 25% only reduces average efficacy to 0.38, a ∼15% reduction. Second, the increased efficacy in seropositive vaccinees produces a stabilizing effect: if epidemics become large, the vaccine performs better thus driving epidemics smaller, while if epidemics have been small, overall efficacy decreases, permitting larger epidemics. These offsetting effects make population-level effectiveness relatively robust.

Our ability to forecast vaccination impact is primarily limited by the current uncertainty regarding whether and how vaccine efficacy wanes over time and how vaccine efficacy is affected by prior infection. Nevertheless, our model provides a useful perspective on how vaccine properties and strategic choices affect the relative size and severity of projected epidemics. The long-term effectiveness of a strategy is a function of vaccination effort, the efficacy and duration of vaccine-induced immunity, and the interaction of these factors with the generation of natural immunity through the underlying transmission process. A Dengvaxia-like vaccine will be a great advance in dengue control in the short-term, but will not be a complete, long-term solution: even under the most optimistic scenarios, our model suggests vaccination alone cannot eliminate dengue in Yucatán. Many different dengue mitigation strategies are actively being developed [50–52], and in the long term these may supplement or replace Dengvaxia. As the vaccine is deployed to control epidemics in the near future, development of additional mitigation strategies should continue alongside further study of vaccine performance, to provide the data needed to inform new strategies and, potentially, identify one capable of eliminating dengue.

## Supporting Information

S1 Text Further details on synthetic population, transmission model, parameterization, effect of climate change on results, and prediction intervals for results. (PDF)
