# Supplementary material for: Projected Impact of Dengue Vaccination in Yucatán, Mexico
Source: PLoS Negl Trop Dis. 2016 May 26;10(5):e0004661. doi: 10.1371/journal.pntd.0004661 (PMC4882069; doi:10.1371/journal.pntd.0004661)
Supplement: S1 Text — (PDF) [file pntd.0004661.s001.pdf]

Supporting Information

List of Figures

A Seasonality of model and empirical data 10
B Possible mosquito movements 11
C Simulated mosquito lifespan 12
D Waning vaccine models 14
E ABC-SMC parameter distributions 21
F ABC-SMC metric distributions 22
G Climate change sensitivity 25
H Case prediction intervals (PIs) for durable vaccine 27
I Cumulative case PIs for durable vaccine 28
J Annual effectiveness PIs for durable vaccine 29
K Cumulative effectiveness PIs for durable vaccine 30
L Annual effectiveness PIs for waning vaccine without boosting 31
M Cumulative effectiveness PIs for waning vaccine without boosting 32
N Annual effectiveness PIs for waning vaccine with boosting 33
O Cumulative effectiveness PIs for waning vaccine with boosting 34
P Fitting period (detail) 35

List of Tables

A Relative pathogenicity inputs 3
B Reported dengue cases from Yucatán, Mexico, 1979–2013 4
C Summary of model parameters 15
D Fitted epidemic model parameters 16
E Serotype introduction fitting 19
F Metrics used for epidemic model fitting 23

S1 Dengue Model

S1.1: In Mosquitoes When exposed to dengue by infecting bites, occurring at rate β_{HM}, on infectious humans, susceptible mosquitoes begin incubation. When a mosquito is infected, we randomly draw an extrinsic incubation period (EIP) from a log-normal distribution [1], with μ parameter determined from a temperature time series (see Section S3). Once the EIP passes, the mosquito becomes infectious and remains infectious until death.

S1.2: In Humans When exposed to dengue by potentially infectious mosquito bites, occurring at rate β_{MH}, subsequent infection probability depends on the person's previous infection and vaccination history. If they have been previously exposed to that serotype or are within the cross-immunizing window from a previous infection, then they will not be infected. Vaccination may provide a benefit, depending on vaccine parameters and the individual's vaccination history (see Vaccination, Vaccination). Maternal antibodies may also influence infection probability and the likelihood of severe disease (see next section).

If infected, people undergo an incubation period, Δ_E, then an infectious period, Δ_I, during which they may transmit the infection to uninfected mosquitoes via bite events and which depends on their disease outcome (asymptomatic, mild, or severe disease). Symptomatic individuals may experience mild or severe disease, which affects the probability that a case is reported, and the duration of Δ_I, but does not affect the probability of infecting a susceptible mosquito if fed upon. Asymptomatic versus symptomatic outcomes also affect dynamics in that symptomatic people may withdraw (stay home) instead of going to work or school. During each day of the symptomatic period individuals withdraw with probability 0.5. Once individuals have withdrawn, they remain home until no longer symptomatic.

S1.3: Pathogenicity and Severity Pathogenicity is the probability an infection produces symptoms. In our model, the reference pathogenicity is for secondary infections of with DENV1, and this is a fitted parameter. For other infections, we use relative-risk estimates for serotype-specific factors and number of past exposures to dengue.

For the serotype-specific factors, we used estimates of reported cases per 1000 infections (medians for fixed-duration cross-protection in Table 2 of supplement in [2]), and the observed proportions of mild and severe disease by serotype (Table 4 of [3], using all-years; DF versus sum of all DHF and deaths). For serotype i, the reported cases, C_i, relate to the pathogenicity, ρ_i, the mild fraction, f_i, and the mild and severe expansion factors, EF_m and EF_s by

$$\frac{C_i}{C_1} = \frac{\rho_i}{\rho_1} \frac{\frac{f_i}{EF_m} + \frac{1-f_i}{EF_s}}{\frac{f_1}{EF_m} + \frac{1-f_1}{EF_s}}$$

Using these values, we calculated the relative-risk compared to infection by DENV1, $\frac{\rho_i}{\rho_1}$, based on the sample parameter values for the expansion factors and values of (C_i, f_i) from the literature; see A.

For previous exposure factors, we divide infections into primary infections (no previous dengue exposure), secondary infections, and post-secondary infections. For secondary infections, pathogenicity is a fitted parameter. For post-secondary infections, the relative-risk of pathogenicity is fixed at 0.1. Primary infections, however, depend on age.

For primary infections, newborns (age 0 individuals) may have maternal antibodies. Whenever newborns are exposed to an infectious bite, we randomly draw an individual

Serotype	C_i	f_i
DENV1	1.20	353/1223
DENV2	0.99	176/1477
DENV3	1.00	397/1542
DENV4	0.38	95/480

Table A. The estimated number of reported cases per 1000 infections, C_i [2], and observed proportion of mild disease, f_i [3], by serotype.

from the females in their household of child-bearing age (15–45 years old) to identify a "mother". If that mother has a past dengue infection, then the infant may have maternal antibodies and we draw an age in months for the infant uniformly between 0 and 11. We treat maternal antibodies as cross-immunizing in months 0–2, disease-enhancing (*i.e.*, like a second infection) in months 3–8, and as having no effect at 9+ months. For all other ages, we use a step-wise approximation of the curve from [4] for pathogenicity relative-risk. In our model, age is only a factor for pathogenicity of primary infections, in contrast to the model described in [5].

To determine case severity, we fit a parameter for the probability of severe (*i.e.* DHF, DSS, or death) disease in secondary cases, fit relative-risk of primary cases (compared to secondary cases) that is constrained to be less than 1, and assume a fixed relative-risk (to secondary cases) of 0.2 for post-secondary cases.

S1.4: Dengue Introductions Empirically and in the model, local dengue transmission generally ceases during the winter and spring, which is relatively dry and cool (Fig. A, panel D). It is possible for transmission chains to persist, but improbable because $R_0 < 1$ from late fall to early spring. To continue to have outbreaks in our model, we re-introduce dengue from an unmodeled external source (representing, *e.g.*, travelers that import it from a distinct human population, zoonotic introductions), similar to what may happen in the real system. This external source may also present novel serotypes to the population. We model these introductions as random exposures in the human population, and they may be resisted if an individual is vaccinated or has been previously infected.

The daily number of people exposed is sampled based on a fitted parameter, λ_E: # exposed per circulating serotype $\sim \text{Pois}(\lambda_E)$ (see Section S5). This rate is flat over time: it does not vary by year, nor seasonally over the course of a year. However, since it is the rate per circulating serotype, the total introduction rate is higher in years with more circulating serotypes. When an exposure occurs, it is randomly assigned one of the circulating serotypes. The serotypes being introduced depends on the presence / absence time series for each serotype (see next section). If there are no serotypes circulating in a given year, then there are no introductions.

S1.5: Serotype of Introductions Historical case data for Yucatán (Table B) indicates that serotypes are present (runs) and then absent (gaps) in streaks [6]. Before each simulation begins, time series are independently constructed for each of the four serotypes. For the fitting period, we introduce the serotypes in the years in which they were observed. For historical periods with no recorded data and the forecast period, time series are generated for each of the four serotypes according to separately fit geometric distributions (see Section S5). In a given year there may be any combination of serotypes 1–4 introduced, including no serotypes.

Year	Mild Cases	DHF/DSS Cases (%)	Total (per 100k)	Serotypes 1	2	3	4
1979	4234	0 (0.0)	4234 (409.8)	■	□	□	□
1980	4672	0 (0.0)	4672 (439.2)	■	□	□	□
1981	3377	0 (0.0)	3377 (308.8)	■	□	□	□
1982	1412	0 (0.0)	1412 (125.7)	■	□	□	□
1983	643	0 (0.0)	643 (55.7)	■	□	□	□
1984	5486	9 (0.2)	5495 (464.3)	■	□	□	■
1985	193	0 (0.0)	193 (15.9)	■	□	□	□
1986	34	0 (0.0)	34 (2.7)	□	■	□	□
1987	15	0 (0.0)	15 (1.2)	■	□	□	□
1988	356	0 (0.0)	356 (27.3)	■	□	□	□
1989	2	0 (0.0)	2 (0.2)	■	□	□	□
1990	8	0 (0.0)	8 (0.6)	■	□	□	□
1991	352	0 (0.0)	352 (25.1)	■	■	□	□
1992	22	0 (0.0)	22 (1.5)	■	□	□	□
1993	29	0 (0.0)	29 (2.0)	■	□	□	□
1994	674	6 (0.9)	680 (44.8)	■	■	□	■
1995	65	4 (5.8)	69 (4.4)	■	■	□	□
1996	620	30 (4.6)	650 (41.2)	■	■	■	■
1997	5366	163 (2.9)	5529 (346.2)	■	■	■	■
1998	36	0 (0.0)	36 (2.2)	□	□	□	□
1999	43	0 (0.0)	43 (2.6)	□	□	□	□
2000	0	0 (NA)	0 (0.0)	□	□	□	□
2001	252	35 (12.2)	287 (17.0)	□	■	■	□
2002	749	197 (20.8)	946 (54.9)	■	■	□	□
2003	20	6 (23.1)	26 (1.5)	□	□	□	□
2004	51	6 (10.5)	57 (3.2)	□	■	□	□
2005	123	39 (24.1)	162 (8.9)	■	■	□	□
2006	465	162 (25.8)	627 (34.0)	■	■	■	□
2007	1472	389 (20.9)	1861 (99.3)	■	■	■	■
2008	573	148 (20.5)	721 (37.9)	■	■	□	□
2009	2102	1110 (34.6)	3212 (166.6)	■	■	□	□
2010	1707	810 (32.2)	2517 (128.7)	■	■	□	□
2011	4040	2092 (34.1)	6132 (309.2)	■	■	□	□
2012	10503	2497 (19.2)	13000 (646.7)	■	■	□	■
2013	8023	914 (10.2)	8937 (438.6)	■	■	■	■

Table B. Reported dengue cases from Yucatán, Mexico, 1979–2013 [6]. ■ indicates serotype was reported, □ indicates not reported.

S1.6: Seroprevalence in Yucatán We compare age-specific seroprevalence in year 2014 of the model with a cross-sectional serological survey performed in 2014 in Mérida (main text, Fig. 4) [7, 8]. In that study, serum samples were collected from a random sample of the population aged from one month to 65 years old who lived in Mérida, with a particular focus on children (aged 2–10). The adults were sampled from healthcare facilities in the study area and children were sampled from the general population and schools.

The age-group-specific prevalence of pre-existing antibody-mediated immunity to any of the dengue viruses was defined as the proportion of the sampled individuals in the age group whose serologic specimen is positive to any serotype using Panbio Dengue IgG Indirect ELISA (titer > 0.10). We computed the exact binomial confidence intervals (95%) for these prevalence estimates.

S2 Synthetic Humans

Individuals belong to a household, may travel to a school or workplace during the day, and have an age and gender. These attributes do not change, thus population demographics are static. The only aspect of the population that is not static is immune profile.

For simulations of many years of dengue epidemics, which are necessary to establish long-term dynamics and population-level patterns of immunity, assuming a static population is obviously imperfect. A demographic model of changing population structure, formation of new households and shifting spatial distribution (*e.g.*, due to urbanization) could be more realistic, but it would also be difficult to parameterize and validate, and to reproduce for other populations of interest in future research. The current approach avoids the challenges of modeling past and future demography across varying regions, for which little (or no) data are available.

S2.1: Households The Mexican state of Yucatán is subdivided into 106 municipalities. To determine the number of households within these municipalities, we divide the municipality population by the expected household size for that municipality, based on the 2010 Mexico census [9] and Integrated Public Use Microdata Series, International (IPUMS) data [10]. IPUMS characterizes households by number of residents, their ages, genders, and employment/student status. Individual households were sampled from the IPUMS household distribution for each municipality.

Using boundaries from DIVA-GIS [11], households are located by pixel (resolution of ~500m) within each municipality, proportional to light output recorded by nighttime satellite imagery [12] (main text, Fig. 1). Within pixels, households are located uniformly randomly. The satellite images are composite images taken on nights with no moonlight. The composite images do not contain clouds, but some background noise is present, for example from reflected star light. To prevent the placement of households in regions that are unpopulated, we subtracted from the entire region the average luminosity of an unpopulated region in western Yucatán, the Petenes-ría Celestún Nature Reserve. All resulting negative values were set to zero.

S2.2: Movement, Schools & Workplaces During the day, people may leave home and go to work or school, according to their IPUMS employment/student status and age (main text, Fig. 1). Individuals who are retired, disabled, or unemployed stay home.

Data from the Mexican National Statistics Directory of Economic Units (DENUE) [13] specifies approximate number of employees and workplace postal code for workplaces. Postal codes for each school are provided by the Secretary of Education, Yucatán (SEGEY) [14]. Yucatán contains 509 postal codes, with at least one workplace reported for 236 of them, and schools reported for 210. Number of employees per school is calculated based on the number of students who attend (see below) and a student:teacher ratio of 28 for Mexico in 2012 [15]. Workplaces and schools in the model are placed uniformly randomly within their corresponding postal codes. There are 95,560 workplaces and 3402 schools in the model.

People are assigned to daytime locations according to the rules below. Rules are listed in order of precedence.

- Everyone with an employment status code that is a student code goes to a school.
- Everyone with an employment status code that is a work code goes to a workplace.
- Children under 5 years stay home.
- Children age 5–11 years go to school.

- Remaining individuals (*i.e.* those with disabled, retired, or unemployed codes) stay home.

Students attend the closest school. We use a censored gravity model for employment: workers select from the 1000 nearest workplaces with probability proportional to workplace size over Euclidean distance to the workplace squared. These schools or workplaces are static for individuals, just like their households, and they visit them every day. The only change to this behavior is if they have a symptomatic infection, in which case they may stay home (see Section S1), and they resume their daily movement once their infectious period ends.

S2.3: Immunity & Aging Immunity in the population is established by simulating 100 years of dynamics before assessing baseline dynamics or introducing interventions. An initial population is created with a modest level of immunity by assuming a probability of past infection corresponding to a homogeneous 1% annual attack rate per serotype. We use this approach rather than a fully susceptible initial population in order to avoid huge early epidemics that are time consuming to model and are not meaningful, as no data are available from this time period for comparison. The population is exposed to random dengue introductions for 77 years, followed by a 23 year period with reduced mosquito populations and introductions (see Time), followed by 35 years of random introductions.

These time periods are based on an assumed stable history of regular dengue epidemics in the Yucatán, interrupted by successful elimination (via intense vector control with DDT) that lasted for approximately two decades, ending in 1978. Because the maximum age in the synthetic population is 100 years, we choose 100 years for the historical period so that the population has completely turned over before the fitted period and the majority of the population has lived in a period of relatively stable exposure.

We model aging by transferring the complete disease and immune state from younger to older individuals annually. For each person, the closest person who is one year younger "donates" their immune state. We considered an alternative approach using a censored gravity model: the donor was selected from the closest 100 individuals that were one year younger, with probability proportional to the inverse squared distance between recipient and donor. However, this approach produced no apparent difference in dynamics and was substantially more computationally demanding. Newborns (age 0 individuals) have no prior exposure, but may have maternal antibodies present (see next section for details) contributed from a female in their household.

We "age" the entire population on day 99 of the Julian calendar, which corresponds to April 9 of non-leap years. This is approximately the middle of the inter-epidemic period for Yucatán. By aging all individuals during the inter-epidemic period, we minimize the side effects of changing the spatial distribution of infections and immunity on any on-going chains of transmission. This approach substantially reduces the computational complexity of the model relative to individuals having birthdays throughout the year, and it has only a modest effect on transmission, seen as a discontinuity in the red simulated cases curve in early April (main text, Fig. 2).

S3 Synthetic Mosquitoes

S3.1: Biting Recall that some people change locations daily between home and school or work, while some remain home. This change occurs at fixed times every simulated day.

We use the female biting behavior in the rainy season in Thailand to estimate relative preference for biting while people are at home (night) versus their daytime locations: we assume 8% of biting takes place between sunrise (7 a.m.) and 9 a.m., 76% takes place between 9 a.m. and 5 p.m; and 16% takes place between 5 p.m. and sunrise [16]. We define the work/school day as 9 to 5, thus there is a 24% preference for dusk-night-dawn biting, and 76% for daytime biting.

For each location, we calculate the biting-time-preference weighted total human population, n, and the preference weighted infectious human population, v, from the biting preference and time-dependent human populations. From n and v, we calculated the weighted fraction of bites that expose mosquitoes to infection, f_v:

$$\begin{aligned} n &= 0.76 n_{\text{day}} + 0.24 n_{\text{night}} && \text{(A)} \\ v &= 0.76 v_{\text{day}} + 0.24 v_{\text{night}} && \text{(B)} \\ f_v &= \frac{v}{n} && \text{(C)} \end{aligned}$$

We use f_v with the effective biting rate, β_{HM}, to determine to determine the number of newly infected mosquitoes: $N \sim \text{Binomial}(m, \beta_{HM} f_v)$, where m is the number of susceptible mosquitoes. The susceptible mosquito population at a location is the location's seasonally adjusted capacity minus the number of tracked mosquitoes (those that can potentially infect people) currently at that location. This is in contrast to the previously published model description in which the susceptible mosquito population was the location's capacity minus the number of infected mosquitoes generated at that location that are still alive [5]. The infecting serotype is drawn from the serotype frequencies at that location, also time-weighted by biting preference.

Infected mosquitoes are represented as mobile, individual agents that are generated in locations with infected humans. Infectious mosquitoes make an infectious bite (which may be resisted via immunity) with probability β_{MH} each day; this parameter represents the product of biting frequency and mosquito-to-human transmissibility, and β_{MH} can be understood as the daily probability of transmission per infectious mosquito in a fully susceptible human population. Infectious bites occur either during the day or night, proportional to the population during those times weighted by biting preference:

$$\Pr\{\text{day bite}\} = \frac{0.76 n_{\text{day}}}{0.76 n_{\text{day}} + 0.24 n_{\text{night}}} = 1 - \Pr\{\text{night bite}\} \qquad \text{(D)}$$

Once bite timing is drawn, the bitten individual is randomly drawn from those present during that interval, with replacement if there are multiple infectious biting mosquitoes.

S3.2: Seasonality & Spatial Distribution Seasonality in the model is represented by daily varying EIP and mosquito population size. For both temperature and precipitation data informing these curves, values for February 29 were discarded in order to make all years have exactly 365 days.

EIP, or time between a mosquito taking up virus from a host until the mosquito is able to transmit the virus, is highly sensitive to ambient temperature [1]. Chan and Johansson (2012) found that the distribution of EIP (in days) for dengue at a constant temperature (in Celsius) is best fit by a Log-Normal ($\ln \mathcal{N}$) distribution:

$$EIP \sim \ln\mathcal{N}\left(\mu = e^{2.9-0.08T}, \sigma = \sqrt{\frac{1}{4.9}}\right) \tag{E}$$

However, the real environment has a seasonally varying temperature, not a constant one, so we must integrate to obtain the correct expected EIP as a function of time of year. We can do so by calculating the incubation *rate* associated with the expected EIP ($\overline{EIP} = e^{\mu(T)+\frac{\sigma^2}{2}}$) in a given interval δt_i with temperature T_i:

$$\frac{1}{\overline{EIP}(T_i)} = e^{-\mu(T_i)-\sigma^2/2}$$

Then the EIP forward from time interval k is

$$EIP_k = \sum_{i=k}^{j} \Delta t_i \quad \text{s.t.} \quad \sum_{i=k}^{j} \frac{\Delta t_i}{\overline{EIP}(T_i)} = 1$$

We used an input time series of hourly (*i.e.* $\Delta t_i = 1/24$) temperatures for Mérida, and linearly pro-rated the final interval to obtain $EIP_k = 1$ exactly. This time series was not complete for the fitting interval (missing ~8.6% of hourly temperature readings), so we estimated missing values using temperature data from Miami, USA, which is climatically similar to Mérida and is more complete (~0.02% missing). We related the temperatures between Miami and Mérida using ordinary least squares regression, which in turn identified high standardized residual ($|\text{SR}| > 5$) in a modest number (73 out ~334k non-missing points, or 0.02%) of points in the Mérida time series. We discarded these points, refit, and used the resulting model to fill in missing temperatures. We used the hourly temperatures to calculate hourly incubation rates, which we then integrated over as described above to obtain the EIPs on an hourly basis. We found the weighted average EIP for each day using the day versus night biting preferences assumptions (see previous section). In the simulation, this expected EIP is used to calculate μ for the Log-Normal distribution on a particular day, which we draw from when infecting mosquitoes.

Mosquito population size also varies seasonally. The number of susceptible mosquitoes per location is dependent on a daily scaling factor, and a location-specific baseline capacity.

Daily scaling factors are based on precipitation history (Fig. A, panel B) in Mérida. Using a climatological dataset from NOAA, we calculated the proportion of years that each day had precipitation. We then fit a cubic smoothing spline, lagged by 7 days to accommodate the pre-adult life stages of *Ae. aegypti* [17]. We normalize the spline to a maximum of 1.

At the beginning of the simulation, each location is assigned a maximum capacity sampled from an exponential distribution with a fitted mean, $1/M_{peak}$ (see Section S5). We multiply the sampled peak for a given location by the seasonal scaling series to obtain the daily capacities for that location.

S3.3: Movement Infected mosquitoes may change locations (*i.e.*, houses, workplaces, and schools) on a daily basis (with probability 0.15; [5]). See Fig. B for a visualization of all the locations. If mosquitoes do move, they select from adjacent locations proportional to the inverse squared distance, where the distance is the Euclidean distance between the source and potential destination based on their latitude and longitude.

Adjacency among the approximately 480k locations is determined using a filtered Delaunay triangulation [19]. We preclude long distance movement by removing edges

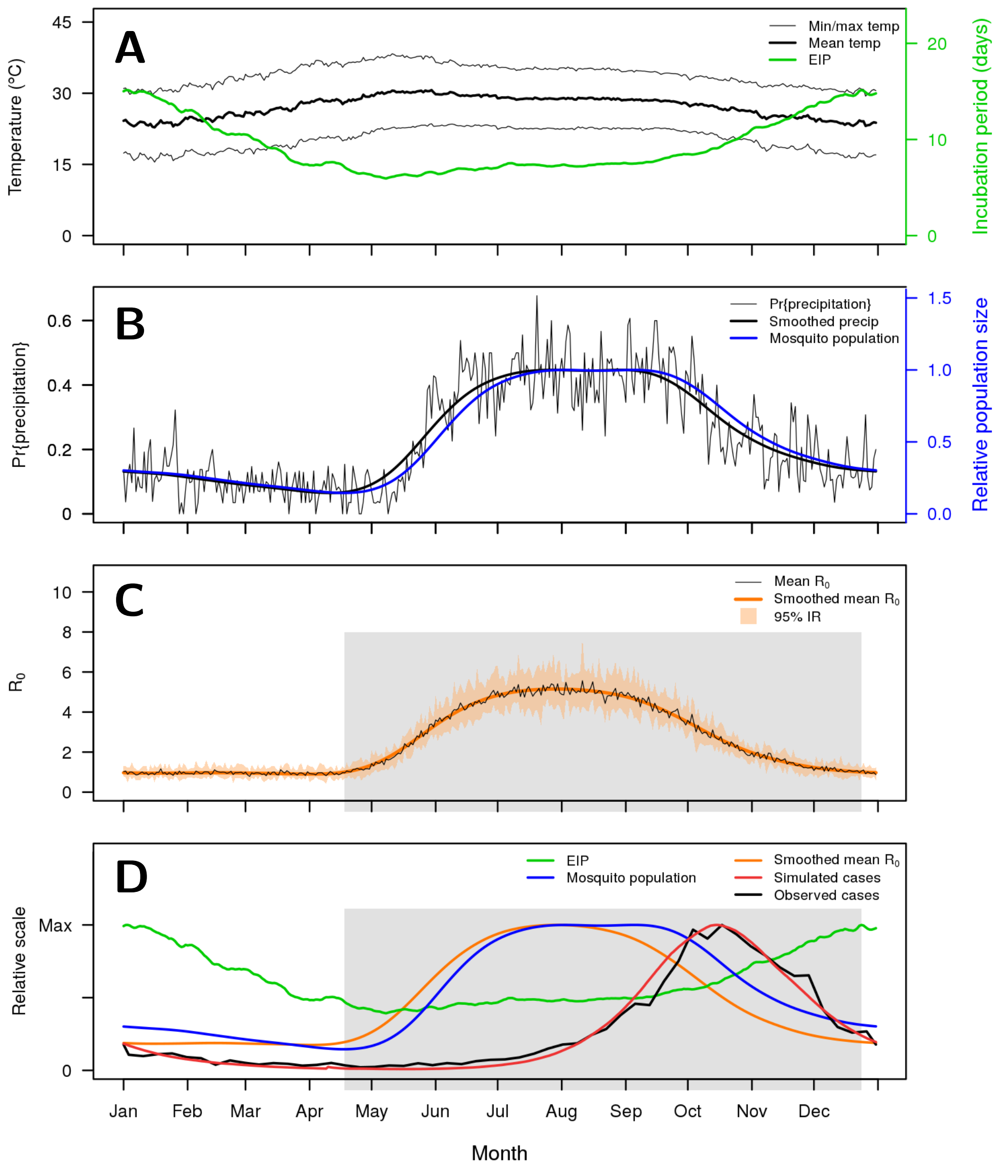

Figure A. Seasonality in the model is driven by daily changes in the extrinsic incubation period (EIP) and mosquito population size (see Section S3). (A) Resulting daily EIP. (B) Smoothing spline (bold black) fit to daily probability of precipitation, then lagged 7 days to account for egg-to-adult mosquito maturation time (blue). (C) R_0 estimated by averaging the number of secondary cases resulting from a single introduction to a fully susceptible model, for each day of the year for all 100 parameter combinations in the posterior, with 10 replicates for each parameter combination. Shaded orange region is the 95% interquantile range (IR); shaded gray regions indicate time of year when R_0 exceeds 1. (D) Seasonal changes in EIP and mosquito population size result in seasonality of simulated dengue cases that matches the reported case data for Yucatan, Mexico (1995–2011). Climatological data for Mérida, Mexico (1979–2014) provided by NOAA [18].

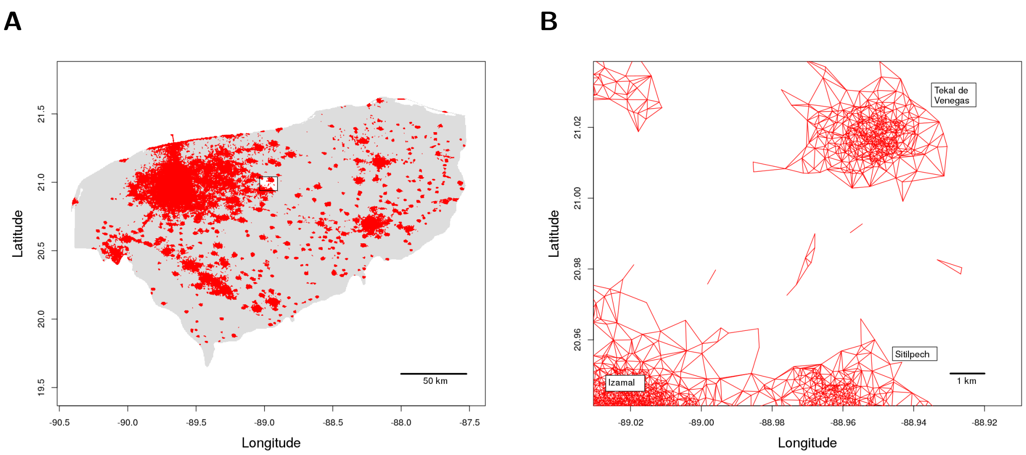

Figure B. (A) Delaunay triangulation of Yucatán locations, with edges more than 1 km removed. (B) Detail, indicated by rectangle in (A).

with a haversine length greater than 1 km. As a consequence of traveling along this network, mosquitoes will only travel short distances in densely populated areas and longer distance in sparser areas, which is consistent with an empirical study [20].

S3.4: Lifespan Mosquitoes must make at least two blood feeding attempts to transmit dengue between human hosts, separated in time by at least the EIP. The lifespan of the mosquito thus influences dengue transmission. We assume that mosquitoes have a lifespan determined by a logistic hazard [21]:

$$H(t) = \frac{ae^{bt}}{1 + \frac{as}{b}(e^{bt} - 1)} \tag{F}$$

where $a = 0.0018$, $b = 0.1416$, and $s = 1.0730$ are the estimated coefficients for caged female *Ae. aegypti* (Fig. C). By discretizing this hazard and imposing a maximum age of 60 days, we can compute the corresponding cumulative survival probabilities, and then the steady state discrete age distribution of living (female) mosquitoes. When a mosquito is infected with a particular dengue serotype, we draw twice from this distribution to determine (1) how old the mosquito is when it is infected and (2) given that biting age, at what age it will die. This approach is an approximation, since the correct age-of-infection distribution depends on historical local dengue prevalence, but tracking that and accounting for mosquito movement would be substantially more computationally demanding.

When a mosquito is infected, we draw an EIP from that day's distribution. If her remaining lifetime exceeds that EIP, we then track the mosquito. Once the EIP passes, the mosquito becomes infectious until it dies. Bites by that mosquito expose humans to dengue infection.

While wild mosquitoes in Yucatán may have longer or shorter lifespans than described by Eq. F, the overall dengue model dynamics are calibrated using these estimates, so differences can be absorbed into other parameters. For example, if wild mosquitoes in Yucatán actually live longer than these estimates and all other parameters are held constant, we would expect the model to underestimate dengue incidence. We can maintain the correct expected dengue incidence, however, because we are fitting the number of biting mosquitoes. In this example, the mosquito population size parameter could increase to maintain a realistic force of infection.

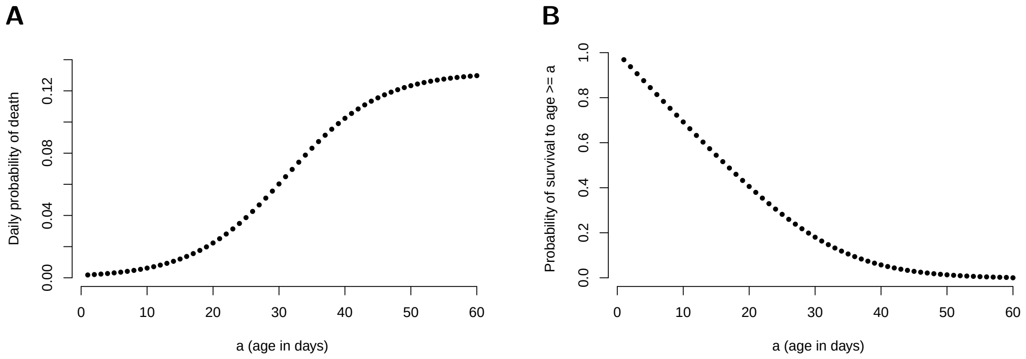

Figure C. (A) Female *Ae. aegypti* mortality, from [21]. (B) Survival cumulative density derived from the hazard.

S4 Vaccination

The effectiveness of vaccination strategies is assessed by contrasting the forecasted dengue burden with and without vaccine deployment over a 20 year period (see Time) corresponding to 2014–2033. We assume the vaccine gives partial, *i.e.*, leaky, protection, and that vaccination does not affect infectiousness when breakthrough infections occur. All vaccinations occur on day 100 of the Julian calendar, which approximately corresponds to the epidemic nadir (see Fig. A).

S4.1: Efficacy We assume vaccine efficacy (VE) consistent with the the phase III trial results for the Sanofi-Pasteur vaccine in Latin America [22, 23], where the overall VE by serotype is from the intent-to-treat analysis for the trial. In that trial, the estimated overall VE, ignoring serotype, was twice as high for those who were antibody-primed for any dengue serotype (*i.e.*, those previously infected), compared to those who were antibody-naïve (never previously infected). These VE estimates for antibody-primed and antibody-naïve individuals per serotype are not available from the vaccine trial results, but we produced the approximate values (see main text, Table 1), by assuming the antibody positive rate in the vaccine trial participants was $\rho_{\text{sero}+} = 60\%$ when they were vaccinated. Specifically, we used the following relations to calculate VE by serotype:

$$
\begin{aligned}
VE &= VE_{\text{sero}+}\rho_{\text{sero}+} + VE_{\text{sero}-}(1-\rho_{\text{sero}+}) \\
\frac{VE_{\text{sero}+}}{VE_{\text{sero}-}} &= 2
\end{aligned}
$$

Vaccinees retain perfect protective immunity against serotypes they were previously infected with. Among individuals with an infection history (“primed”) who are challenged with a novel serotype, the modeled vaccine is highly effective against serotypes 3 and 4 and moderately effective against serotypes 1 and 2. Among naïve individuals, vaccine efficacy is lower for all four serotypes, but protection against serotypes 3 and 4 are still higher than against 1 and 2. When originally naïve vaccinees experience a breakthrough infection, they subsequently have the elevated VE, as if they had been primed at the time of vaccination.

S4.2: Routine We consider routine vaccination of three different target groups: 2, 9, or 16 year olds. The number of vaccine doses is held constant across target groups, resulting in variable coverage, or the probability that an individual in the target group is vaccinated. We vaccinate 80% of 9 year olds, requiring 30,100 vaccinations (90,300 doses). This number of vaccinations results in 82% and 74% coverage of 2 and 16 year olds, respectively. Coverage decreases with age because age categories do not monotonically decrease in size: there are 37k, 38k, and 41k 2, 9 and 16 year olds, respectively.

Allowing coverage to vary while keeping the number of vaccinations fixed makes it easier to attribute the cause of effectiveness differences between strategies. In particular, it becomes possible to distinguish between the effects of better vaccine efficacy due to vaccinating older, antibody-positive individuals, versus administering more doses.

S4.3: Routine with Catch-up We also consider strategies where routine vaccination is supplemented by a one-time catch-up campaign of all individuals older than the target age, up to and including age 30. As with routine vaccination, coverage is affected by the age distribution, and the expected number of vaccine doses administered is held constant across catch-up scenarios. The vaccination probabilities

for 3–30, 10–30, and 17–30 year old catch-up scenarios are 45%, 60% and 95%, respectively, corresponding to administering 448,500 vaccinations (1,345,500 doses).

S4.4: Waning and Boosting Vaccine-induced immunity may not be durable. To study the effects of a vaccine with waning efficacy, we use four different linear waning models: no waning, and waning with half-lives of 10, 5, and 2 years (Fig. D). When a vaccinated (but otherwise susceptible) person is bitten by an infectious mosquito, vaccine efficacy given waning $VE_{S,w}$ depends on baseline efficacy VE_S, the number of days t since the person was vaccinated, and the lifespan of the vaccine in days, D:

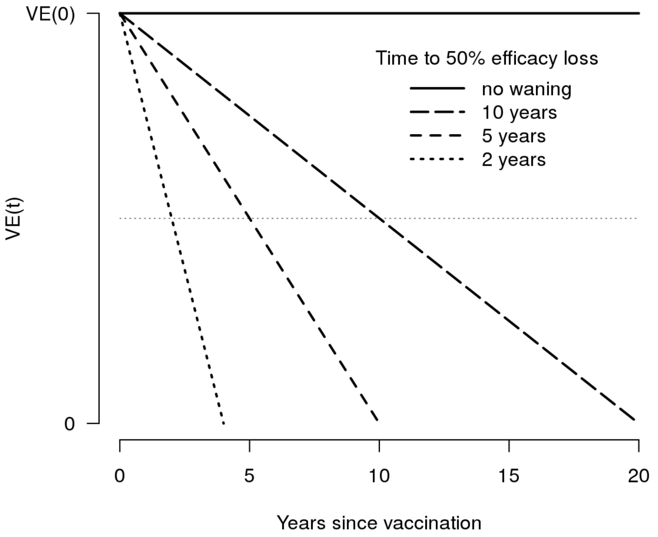

Figure D. Waning vaccine models.

$$VE_{S,w} = \begin{cases} VE_S(1 - \frac{t}{D}) & t < D \\ 0 & t \geq D \end{cases} \tag{G}$$

For scenarios where waning occurs, we consider the effects of booster vaccinations. In all scenarios with booster vaccinations, individuals who were originally vaccinated as part of a routine or catch-up campaign are vaccinated every two years. The efficacy values are the same as those for the initial vaccination.

S5 Parameterization

S5.1: Assumed parameters Some model parameters have been estimated by other studies and would be difficult to identify using the empirical data we have for Yucatán. These assumed parameters are summarized in C.

Parameter	value	source
mean human incubation period, Δ_E	4.8 days	[24]
asymptomatic infectious period, $\Delta_I(A)$	2 days	[24]
mild case infectious period, $\Delta_I(M)$	4 days	[24]
severe case infectious period, $\Delta_I(S)$	6 days	[24]
primary infection pathogenicity RR	varies by age	[4]
daily Pr {withdrawal} when symptomatic	0.5	[5]
duration of cross-immunity	2 years	[25]
biting preference	day: 0.76; night: 0.24	[16]
mosquito mean daily Pr {movement}	0.15	[5]
daily Pr {bite & transmit, M to H}, β_{MH}	0.25	[5]
daily Pr {bite & transmit, H to M}, β_{HM}	0.1	[5]

Table C. Summary of model parameters from literature (see Section S1); M to H is mosquito to human; H to M is human to mosquito.

S5.2: Approximate Bayesian Computation We estimate our six free model parameters (D) using Approximate Bayesian Computation with Sequential Monte Carlo [26, 27] (ABC–SMC). ABC estimates parameters by sampling parameter space, running the model with those parameters, and keeping parameter combinations that produce model results similar (by some quantitative measure) to observed data. However, thoroughly sampling a high-dimensional parameter space may be computationally intractable. Using SMC improves efficiency of ABC by sampling from intermediate distributions (called predictive priors) based on previous ABC results, which focuses the sampling on regions that have produced results that look like observed data.

Quantifying what is meant by saying simulated results are similar to observed data can be challenging. For some models and observations–*e.g.* a disease with stable, predictable annual outbreaks–we might be satisfied reproducing only the mean epidemic size and similarity would reduce to the absolute error in this result. For reproducing more complicated observed data, such as matching different types of data (*e.g.* epidemic size distribution moments and autocorrelation patterns), how to score similarity is less obvious. That is the case here, as dengue in Yucatán has complex interannual dynamics that we would like to be able to reproduce, and we have different types of observations, *e.g.* quantiles on total reported cases, annual autocorrelation, severe case trends, and seroprevalence in a particular population at a particular time. How to get a single distance between the observed data and simulated results is less obvious for this case.

A simple but flawed approach to matching multiple outcomes, or *metrics*, is to calculate the Euclidean distance between model and observed metrics. Of course, metrics may be on fundamentally different scales, so we need a way to normalize scale to avoid giving undue weight to measurements with large units. Next, metrics may be correlated with each other, meaning we may give undue weight to the same kind of information, by including related metrics (*e.g.* mean and median are both measures of central tendency). Additionally, some metrics may be primarily noisy and drown out

Parameter	Prior	Posterior		
		Mean	Median	95% BCI
EF_m	U(10,80)	12.0	11.7	(10.1, 15.0)
EF_s	U(1,5)	4.15	4.23	(2.89, 4.94)
SP	U(0.2,0.8)	0.688	0.705	(0.559,0.783)
SS	U(0.0,0.4)	0.0249	0.0199	(0.00289,0.0650)
$PSSR$	U(0,1)	0.488	0.481	(0.255, 0.727)
$\log_{10}(\lambda_E)$	U(-2, 1)	-0.748	-0.514	(-1.77, -0.151)
M_{peak}	U(20,100)	74.5	74.1	(52.9, 97.1)

Table D. Prior distributions and posterior means, medians and 95% Bayesian credible intervals (BCI) for fitted epidemic model parameters. EF_m and EF_s are expansion factors for mild and severe disease, respectively; SP is the reference (secondary infection with DENV 1) pathogenicity; SS is reference (secondary case) probability of severe disease; $PSSR$ is the relative-risk of severe disease in primary cases; λ_E is the introduction rate (number of exposures per serotype per day, for the entire modeled population); M_{peak} is the mean number of mosquitoes per location, in the month when mosquito populations are largest. See Section S5 for further details.

useful information in others. Partial least squares regression (PLS) addresses these problems [27, 28].

We do not know *a priori* the correlation between model parameters and simulated observed metrics, but we can use PLS to simultaneously orthonormalize both, into a new representation of latent metrics, called *factors* that correspond to centered, rescaled combinations of the original metrics. These factors are uncorrelated and ranked by how much variance they explain in the parameters. We choose a subset of the factors, according to the strength of their relationship with the model parameters. The number of factors used is determined by calculating the predicted residual sum of squares (PRESS) for each possible number of factors by dividing the dataset into training (80%) and testing (20%) partitions. We find the minimum PRESS for any number of factors, and then find the minimum number of factors that is not significantly different ($\alpha = 0.1$) from the minimum PRESS using the Wilcoxon signed-rank test. We repeat this process for each parameter, and the overall number of factors chosen is the maximum of the optimal number for each parameter.

Using the PLS regression corresponding to this reduced set of factors, we transform the empirical observed data, giving us empirical data in the reduced, orthogonal PLS space. We can then calculate the least squares distance between this vector and all of the transformed model outputs, giving us Euclidean distances without the scale or weight issues of the naïve approach. We select a small fraction of the vectors of simulated metrics, corresponding to the smallest distances from the observed values, and use their corresponding parameters as the predictive prior for the next SMC set.

If the model is a useful approximation of reality, and the initial parameter distributions include the realistic values, successive SMC sets will improve the approximations of parameter distributions that reproduce observed metrics. After some number of SMC sets, the parameter distributions cease to improve, at which point ABC-SMC has extracted as much information about the parameter distributions as possible from the metrics.

A more precise description of our ABC-SMC algorithm is the following, based on an approach described in [26], where N is the number of parameter combinations sampled, ρ is the fraction retained in the predictive prior/posterior, and $n = \lfloor \rho * N \rfloor$ is the size of

the predictive prior/posterior:

1. Set the SMC iterator $t = 1$.
   (a) For sample iterator $i = 1, \ldots, N$:
      i. Sample parameter vector $\theta_i^{(1)}$ independently from prior distributions $\pi(\theta)$
      ii. Simulate data $x_i^{(1)} \sim p(x_i^{(1)} | \theta_i^{(1)})$.
2. Calculate PLS model for $\theta^{(t)}$ and $x^{(t)}$
3. Transform observed data and each $x_i^{(t)}$ to new independent, orthogonal metrics using PLS model
4. Calculate Euclidean distance between transformed observed and transformed simulated metrics
5. Set $\Theta^{(t)} =$ best $\theta^{(t)}$, the best n samples, ranked by smallest distance.
6. If $t > 1$ and Θ has converged, stop here. Otherwise:
   (a) Set τ_{t+1}^2 equal to twice the variance of $\Theta^{(t)}$
   (b) For $j = 1, ..., n$:
      i. Set weight $\omega_j^{(t)} \propto \begin{cases} 1/n & : t = 1 \\ \pi(\Theta_j^{(t)}) / \sum_{k=1}^{n} \omega_j^{(t-1)} K(\Theta_j^{(t)} | \Theta_k^{(t-1)}; \tau_t^2) & : t > 1 \end{cases}$
      where $K(\Theta_j^{(t)} | \Theta_k^{(t-1)}; \tau_t^2)$ is a Gaussian perturbation kernel with mean $\Theta_k^{(t-1)}$ and variance τ_t^2, evaluated at $\Theta_j^{(t)}$.
   (c) Normalize $\omega_i^{(t)}$ to sum to 1.
   (d) Set $t = t + 1$
      i. For $i = 1, \ldots, N$:
         A. Choose θ_i^* from $\Theta^{(t-1)}$ with probabilities $\omega^{(t-1)}$.
         B. Sample $\theta_i^{(t)}$ from $Gaussian(\theta_i^*, \tau_t^2)$
         C. Simulate data $x_i^{(t)} \sim p(x_i^{(t)} | \theta_i^{(t)})$.
   (e) Go to step 2.

The weight function described in step 6(b)i favors parameter vectors that have relatively greater density in the prior distribution and lesser density in the most recent predictive prior, thus offsetting a sampling bias that would otherwise be introduced.

The algorithm above differs from that in Beaumont (2010) in that the best $n = \rho * N$ parameter combinations is retained, rather than an absolute number that score better than some threshold ϵ that decreases with each SMC step. Beaumont's approach involves sampling parameter space an indefinite number of times until a fixed number of samples have resulted in a distance less than ϵ. Because we necessarily use an updated PLS model for each SMC step, the meaning of the numerical value of ϵ changes from step to step. Indeed, the dimensionality of the space that ϵ would be applied to is changing. It is also impossible to confidently estimate the running time required for Beaumont's algorithm, which is a common requirement in supercomputing environments that use a job scheduler. If the selected threshold is too strict, it is possible that no parameter vectors would ever be selected, and the algorithm would run indefinitely. Specifying ϵ but setting an arbitrary time limit could result in an undefined predictive prior.

For fitting of the serotype introduction patterns (see below), we use $N = 10^6$ and select the best performing 1% of parameter combinations. More thorough sampling is possible than with the epidemic model parameters, because simulation of the serotype patterns is orders of magnitude faster.

Selecting good ϵ values is a weakness of ABC-SMC methods, requiring prior knowledge of how a particular model will perform for observed data. To usefully pick such a value, we would need to run the model many times to get an idea of what value would provide sufficient samples. By instead selecting a fixed fraction of parameter vectors, we can reliably estimate run times. Due to the computationally intensive nature of ABC, it is appropriate to design the algorithm in a way that is compatible with supercomputing environments.

S5.3: Serotype Introduction Patterns Our model uses a random serotype introduction series for years for which we do not have specific serotype circulation data, namely the historical periods and the forecast. To simulate an arbitrary number of years of dengue epidemics, we need to be able to generate a pattern of serotype introductions that reproduces circulation patterns in Yucatán for the past 35 years. Since we do not know which serotypes were introduced each year, which is what we need as input to our epidemic model, we use detected, serotyped cases as a proxy for the introduced serotypes. While straightforward, this approach confounds introductions with pathogenicity and transmission: a serotype is more likely to have been reported if it caused more cases, particularly more severe disease. Similarly, though R_0 is low during the first fourth months of the year, inter-season transmission chains are still possible, so the last year in a series of observations of a particular serotype may be carry over from the previous season.

All four dengue serotypes have been observed in Yucatán, but they are present with varying runs and gaps: for example, DENV1 was observed from 1987 to 1997 (an 11 year *run*), and then was not observed from 1998 to 2001 (a 4 year *gap*). A plausible biological explanation is that an introduced serotype is able to persist regionally until a threshold of herd immunity is reached, at which point dengue levels may decrease until another serotype is introduced or enough new susceptible people enter the population, via birth or immigration, for the first serotype to circulate again. Consistent with that interpretation–*i.e.* gaps are driven by herd immunity–we assume that the initial delay in appearance of a serotype is not a gap.

We generate random sequences of alternating runs and gaps for each serotype by sampling from geometric distributions (*i.e.* year ranges of $\{1, 2, 3, \dots\}$ with probabilities of $\{p, (1-p)p, (1-p)^2 p, \dots\}$). Runs or gaps of length 0 do not occur. We use eight separate distributions: for runs and gaps, and for each of the four serotypes. We fit the distribution parameters p_{run}, the probability that a run will end in a given year, and p_{gap}, the probability that a gap will end, separately for each serotype using ABC-SMC.

Because the observed data are incomplete (right censored after 2013, missing for 1998–2000 and 2003) the observed mean run and gap lengths, $1/p_{run}$ and $1/p_{gap}$, are expected to be less than the means for the underlying mechanism. We can make the correct comparison by similarly removing information from the simulated series before calculating their means.

When fitting in ABC-SMC, we use Uniform(1,20) priors for all $1/p_{run}$ and $1/p_{gap}$ parameters. We generate a sequence of runs and gaps that we then sum. The duration of the first run d_0, is sampled from the appropriate geometric distribution. Subsequent deviates are sampled from alternating gap and run distributions until the total duration exceeds the relevant duration for the subject serotype (35 years for DENV1, 28 for DENV2, 18 for DENV3, and 30 for DENV4). We then remove the years corresponding to 1998-2000 and 2003, breaking up any runs or gaps that fall there. Finally, any run or

gap length beyond the series limit is also removed. The mean simulated run and gap durations, after imposing breaks in missing years and removing any duration beyond the available data, are the metrics that are compared with the actual observed mean durations from the Yucatán data. For each SMC iteration, we sample 10^6 parameter combinations, and keep the best 1% for the predictive priors and posterior.

Fitting these parameters at the same time as the others might improve the dengue introduction match to observations, since there is an interplay between introduced serotypes and which are observed at what time, as well as inter-serotype dynamics. However, it is not practical because of how much it would increase the complexity of parameter space, and how long it takes to run the epidemic simulation once. Furthermore, the data available from the fitting period may not be particularly applicable to the past and forecast introduction series, given that these may be largely driven by external phenomena that are insufficiently reflected in the fitting period, so a more detailed approach is unlikely to improve forecasts.

The actual observed, simulated observed, and distribution means are reported in E.

	Actual observed mean	Simulated observed mean	Distribution mean ($1/p$)
DENV1 runs	7.25	7.43	13.83
DENV1 gaps	1.00	1.00	2.70
DENV2 runs	3.80	3.90	9.53
DENV2 gaps	3.00	3.05	8.32
DENV3 runs	1.50	1.52	3.09
DENV3 gaps	2.25	2.26	4.92
DENV4 runs	1.60	1.68	2.55
DENV4 gaps	3.80	3.89	8.50

Table E. Mean duration (years) of presence (runs) and absence (gaps) for each serotype. Simulated observed means were generated using the distribution means and then censored as the observed data were censored.

S5.4: Epidemic Model Parameter Estimation The remaining model parameters were estimated using the ABC-SMC procedure described above to identify parameter combinations that produce dynamics similar to the observed data. For "similarity", we consider the following metrics:

- descriptive statistics for annual reported cases per 100,000 people, 1979–2013
  - mean
  - all quartiles
  - standard deviation
  - skewness
  - annual autocorrelation (median crossing rate)
- seroprevalence in Mérida children aged 8–14, 1987
- descriptive statistics for annual reported severe cases, as a proportion of all reported cases, 1979–2013

  - severe proportion: overall fraction of reported cases that were severe
  - β_0, β_1 logistic regression parameters of the trend in severe proportion, given by Eq. H

$$\text{severe proportion for year } X = \frac{1}{1 + e^{-(\beta_0 + \beta_1 X)}} \tag{H}$$

The mean, quartiles, standard deviation, and skewness have the conventional definitions. A median crossing is defined as a sequential pair of years with one year above and one below the whole series median, and the median crossing rate is the number of median crossings per total years considered minus one.

Only limited serosurvey data are available: school children aged 8–14 in two groups, both within the municipality of Mérida, were tested in 1987, and were found to have seroprevalences of 56.3% and 63.7% [29]. We do not know how representative these samples were of the population of all 8–14 year-olds in Mérida, but they are nonetheless valuable in identifying the infection:reported case ratio. We assume an empirical seroprevalence of 60% for 8–14 year-olds in Mérida in 1987. When the simulator reaches April 9, 1987, we tally the seroprevalence for that subgroup of the Yucatán population as one of the metrics.

The parameters fitted to the metrics are:

- EF_m, mild expansion factor: ratio of mild cases to reported mild cases
- EF_s, severe expansion factor: ratio of severe cases to reported severe cases
- SP, secondary pathogenicity: fraction of secondary infections that are cases
- SS, secondary severity: fraction of secondary cases that are severe
- $PSSR$, primary severity:secondary severity ratio
- λ_E, daily mean exposure introduction rate
- M_{peak}, seasonal peak of average mosquito population size per location

Priors for these parameters are described in Table D.

We use AbcSmc to fit these parameters. We ran AbcSmc for a total of 14 SMC sets, with a sample size of 10,000 parameter combinations for each set. To reduce the running time of the fitting procedure, during the first 7 sets we modeled a reduced population of only Mérida residents (840k people, 46% of the Yucatán population). This subset was defined by taking all individuals who live between latitudes [20.847583, 21.076652] and longitudes [-89.752178, -89.504299]. People were reassigned to schools and workplaces within the same region, according to the rules detailed previously (see Section S2). The last 7 SMC sets used the full Yucatán population. The metrics we use are calculated on a per capita basis, so the interpretation of the metrics is insensitive to the size of the modeled population, but the size and spatial structure of the population (*e.g.*, one large city versus that city and the surrounding towns and rural areas) may affect epidemic dynamics. Using a smaller population initially allowed us to focus in on promising regions of parameter space with faster-running simulations, which we then explored more thoroughly with the full population.

We observed during the Yucatán SMC sets that the predictive priors were fluctuating noisily rather than converging: see, for example, the introduction rate λ_E in Fig. E. This is may be due to inadequate sample size; however, sampling substantially more than 10,000 parameter combinations per set would be prohibitive, as each parameter combination takes approximately 4 hours to simulate. Rather than arbitrarily

choosing one of the noisy predictive priors and calling it the posterior, we used all 70,000 parameter combinations and their associated metrics (”All-Y” in Fig. E) to construct a PLS regression. We then defined the posterior by choosing the 100 parameter combinations that best predicted the observed metrics (Fig. F); these are the parameter combinations used to produce all simulation results. In order to reduce dynamic uncertainty (statistical noise), we use each parameter combination with 10 different seeds for the pseudo random number generator, resulting in sample sizes of 1000 for each vaccination scenario. The metrics and their observed and posterior values are in Table F. For both processing SMC sets and defining the posterior, PLS was trained on 80% of the parameter combinations and corresponding metrics and validated against the other 20% to determine the appropriate number of factors, which was 7 for all sets.

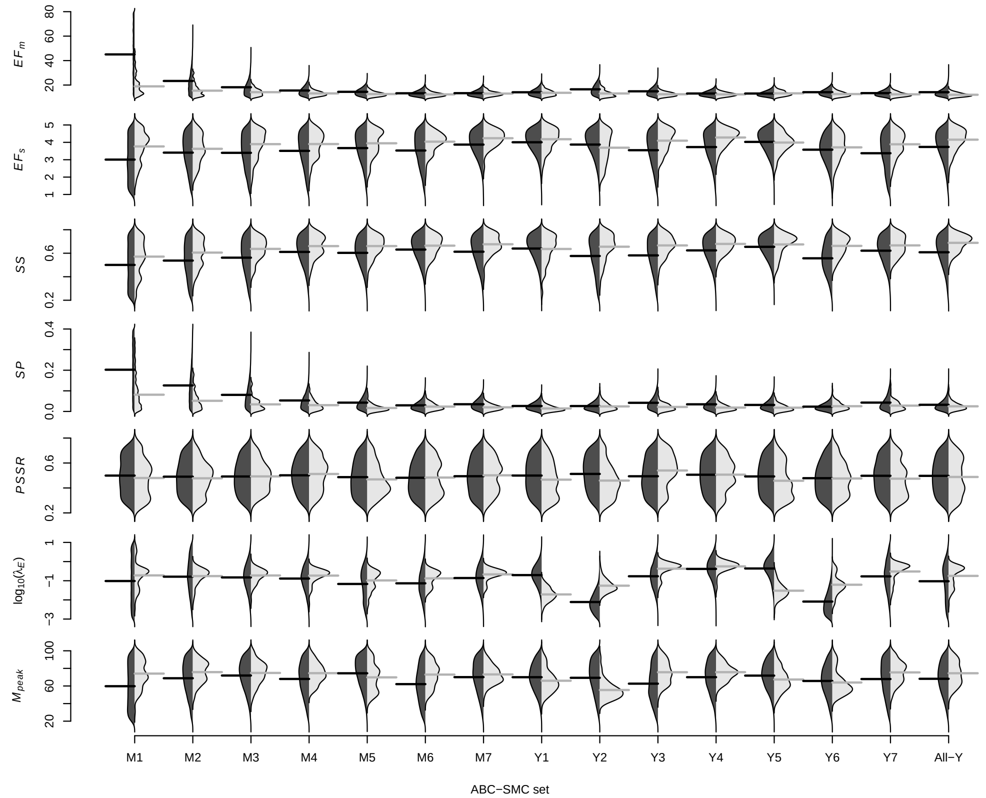

Figure E. ABC-SMC parameter distributions, sampled priors (dark grey) and selected predictive prior or posterior (light grey). M1-7 indicate sets simulated on the Mérida population, which were followed by sets simulated on the Yucatán population (Y1-7). The last column, All-Y is not a normal SMC set, but instead represents all Yucatán parameter combinations simulated in Y1-7 (dark grey) and the global 100 best performing combinations (light grey).

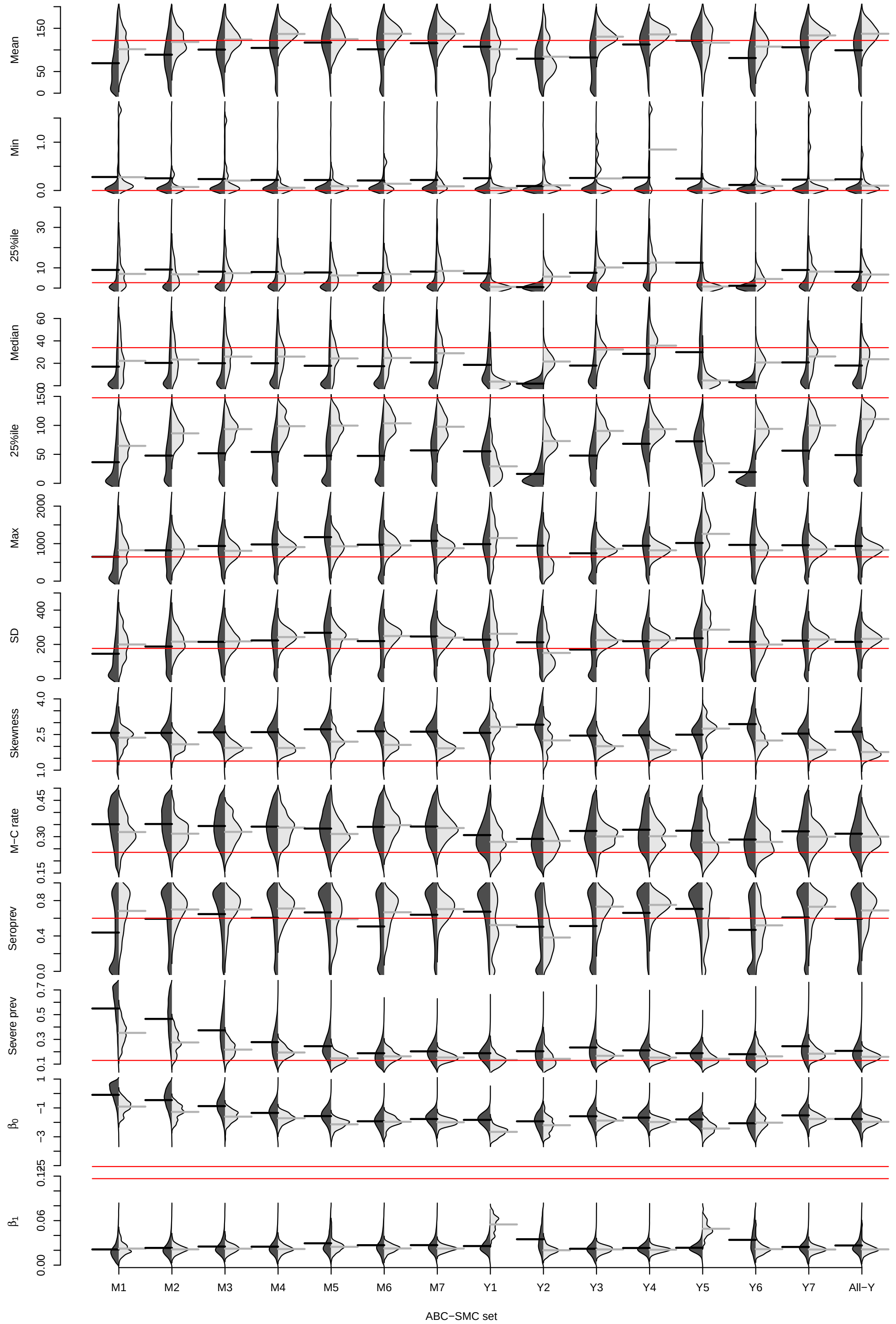

Figure F. ABC-SMC metric distributions, corresponding to sampled priors (dark grey) and selected predictive prior or posterior (light grey). M1-7 indicate sets simulated on the Mérida population, which were followed by sets simulated on the Yucatán population (Y1-7). The last column, All-Y is not a normal SMC set, but instead represents all Yucatán metrics from sets Y1-7 (dark grey) and the global 100 best Yucatán results (light grey). The red lines indicate the observed values for each metric.

Metric	Observed	Posterior		
		Mean	Median	95% IR
mean	122	137	139	(98.0, 172)
minimum	0	0.0219	0.00	(0.00, 0.217)
25th percentile	2.68	6.68	6.39	(0.303, 13.5)
median	34.0	23.6	24.9	(3.67, 39.2)
75th percentile	148	122	120	(77.6, 172)
maximum	647	831	849	(570, 1060)
stdev	177	233	232	(157, 298)
skewness	1.38	1.76	1.72	(1.43, 2.22)
median-crossing rate	0.235	0.300	0.294	(0.206, 0.412)
1987 seroprevalence, 8–14 yo	0.6	0.688	0.729	(0.360, 0.912)
severe prevalence	0.130	0.158	0.160	(0.0923, 0.217)
β_0	-5.06	-1.96	-1.91	(-2.57, -1.53)
β_1	0.117	0.0213	0.0211	(0.0121, 0.0296)

Table F. Observed values and posterior means and medians for epidemic model metrics. Posterior values are for simulations on the Yucatán population after averaging across 10 realizations for each of the 100 parameter combinations that resulted from the fitting procedure. Seroprevalence is for 8–14 year old Mérida residents (subset from the simulated Yucatán population) on April 9, 1987. IR is interquantile range.

S6 Climate Sensitivity

Our mosquito model depends explicitly on temperature and rainfall factors, which are likely to change in the Yucatán over the next two decades. Our main analysis, however, does not consider any long-term trends in climate. To assess sensitivity to climate change, we have considered a scenario consistent with the consensus temperature forecast concerning Yucatán: that the region will become warmer by 0.02 °C per year [30]. We start by inverting the previously calculated daily EIPs into $T_{\text{eff}}(\text{day})$ (the constant temperature corresponding with that day's mean EIP), then create a forecasting time series of $T_{\text{eff}}((day)) + 0.02 * y$, where y is the forecast year. We then recalculate EIPs using the previously described approach, but with the daily T_{eff}s instead.

In general, we expect dengue incidence to increase with warmer climate. Because we assume that EIP decreases with increasing temperature, the likelihood that an infected mosquito will spread dengue before dying increases in turn. Indeed, when long-term warming is included in the model, we see that dengue incidence increases in both baseline and intervention scenarios (Fig. G). However, because the increases in incidence under climate change are approximately proportional to incidence without climate change, vaccine effectiveness remains essentially unaffected.

While we believe the effectiveness results to be relatively robust to alternate climate forecasts, we emphasize that the incidence forecasts will be more sensitive. For example, since temperature has a non-linear effect on EIP, warmer nighttime lows may have a bigger effect on transmission than warmer daytime highs, even if the average temperature increase is the same. Additionally, the only temperature sensitive component of our mosquito model is EIP, but there are a variety of other mosquito life-cycle factors influenced by temperature [31, 32]. Similarly, we did not include any changes in precipitation. An international consensus model of climate change indicates the region will become drier [33], which may have a moderating effect on any increase in force of infection.

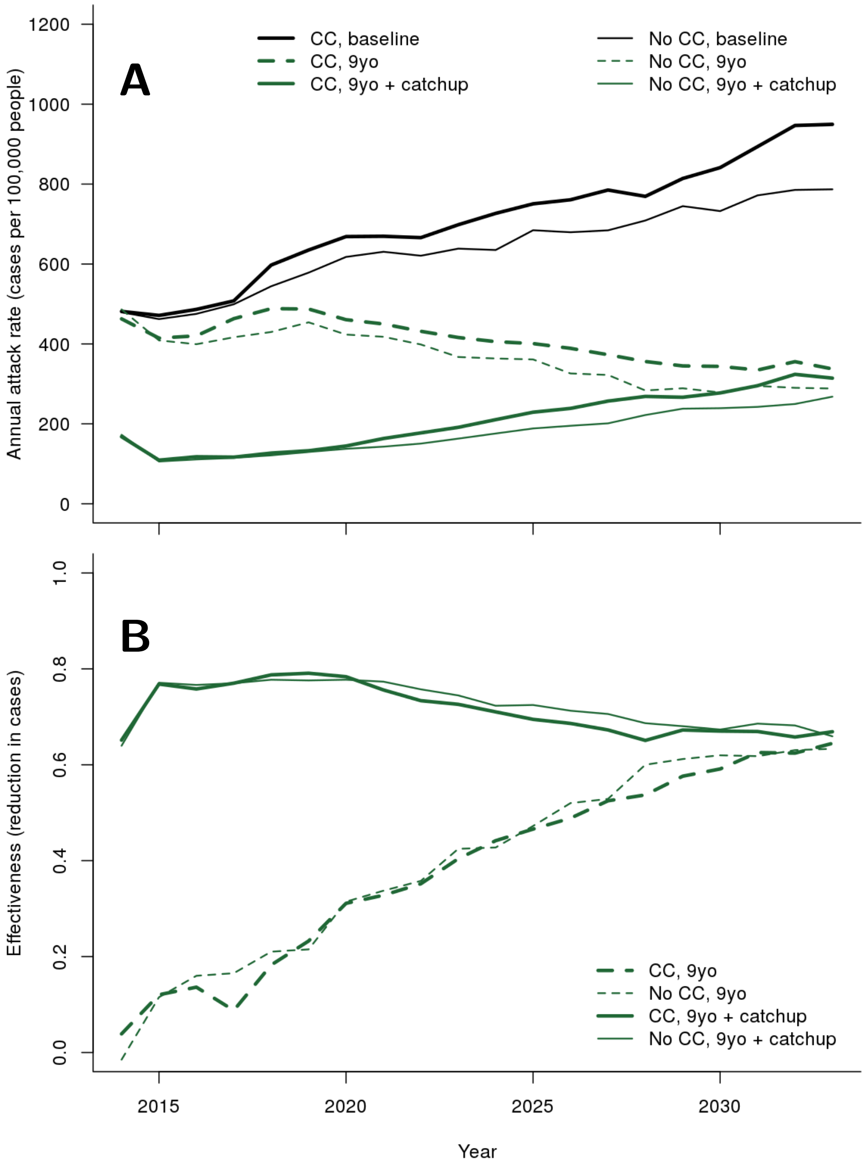

Figure G. Incidence (A) increases as increasing temperature (climate change, CC) drives down EIP. Vaccine effectiveness (B), however, is insensitive to climate change.

S7 Prediction Intervals

In the main results, we reported median outcomes on vaccine effectiveness (V_{eff}) for various scenarios, plotting comparisons across those scenarios within figures. Including prediction intervals (*i.e.* the uncertainty bounds of our forecast) would make those figures difficult to read, so we have included them here by breaking the scenarios into multiple panels. This approach maintains interpretability, but comparisons between scenarios is more awkward and requires substantially more space. We also include cumulative V_{eff} comparisons, since the bounds on annual V_{eff} are not additive when considering the long term intervention effect.

Because we are sampling across several different parameter combinations and replicates derived from ABC, we obtain Bayesian prediction intervals for the results (Figs. H–O). We do this by matching results by parameter set and random seed, calculating V_{eff} (main text, Eq. 1), and then taking quantiles of the resulting distribution at 50% (median), 68% interquantile range ($\sim 2\sigma$ wide on a normal distribution) and 95% interquantile range ($\sim 4\sigma$ wide on a normal distribution). Cumulative V_{eff} is computed similarly, but from the start of the intervention up the relevant year, rather than annually.

Annual V_{eff} shows short-term vaccination scenario performance over moving one-year windows of an intervention, which corresponds to thinking about annual snapshots of the program. This is the during-an-outbreak view: for example, what the general populace or officials in a region undertaking a vaccine program might consider their outcomes compared to neighboring regions without vaccination. For effective programs, they will see lower disease burden in most years. However, in some particular year, an outbreak might occur in the region with vaccination and not in a non-vaccinating neighboring area, simply due to the stochastic nature of introduction of the pathogen, disease spread, and (particularly for dengue) manifestation of disease after infection. In the long view, for an effective intervention, these relatively bad years should be fewer and outweighed by the benefit in most years, so it is also useful to consider cumulative V_{eff}.

When aggregating over many simulated samples of these perspective, robustness is an important consideration in choice of statistic (*e.g.* mean versus median effectiveness), since the distribution of V_{eff} can be highly skewed: it has an upper bound of 1 (at most, all cases can be avoided), is typically between 0 and 1 (*i.e.* the intervention is effective), but can go to negative infinity (*i.e.* when comparing any number of cases in an intervention to 0 cases in the baseline). It can also produce large negative V_{eff} by chance when case counts are generally low, or (in the case of annual effectiveness) when an intervention postpones–but does not prevent–an epidemic from one year into another. Even though the resulting epidemic may be smaller, it may be compared to a small number of cases from the baseline where many people have cross-immunity from the previous year's large epidemic. When these delays are misaligned between many samples, large negative mean V_{eff} appears in many years because the individual large negative V_{eff} observations appear at many different times across different runs.

The net result of these concerns is that mean V_{eff} is not a robust statistic. If we calculated V_{eff} on mean cases (instead of using the cases from each sample and then taking the mean of V_{eff}), this is equivalent to calculating a weighted mean V_{eff} where the weights are the relative number of cases in the baseline. This ignores the magnitude of the large negative V_{eff} values associated with low baseline case counts, and likely corresponds with an intuitive perception of those events in many cases (*e.g.* the non-vaccinating, neighboring region reporting no cases when we reported five is not a huge public health failure). However, it does not properly account for large, negative annual V_{eff} values associated with delayed epidemics in an intervention scenario when the baseline merely had its large epidemic earlier.

Median and quantile V_{eff} values, however, are robust, because they are not sensitive to the skewness of the V_{eff} distribution. The sensitivity to negative V_{eff} also tends to be eliminated when considering cumulative V_{eff}s, since the synchronization of annual epidemics between scenarios becomes irrelevant: only total relative case counts for the considered years matters. Similarly, small outbreak years (and their potential for unimportant, yet large negative V_{eff}) are aggregated with large epidemic years, thus tending to make even net negative V_{eff} small unless the intervention actually harms the population. This corresponds to our intuition about how good (or bad) an intervention is. This robustness is also why forecasted cumulative V_{eff} has decreasing uncertainty: including more years means more time for the trend to emerge above inter-annual variation.

Figures H–O show first the intervals for annual and cumulative cases (without intervention and with routine, durable vaccination, across target ages with and without catchup campaigns), then for annual and cumulative V_{eff} for the routine vaccine, then for annual and cumulative V_{eff} for a waning vaccine with and without a booster program.

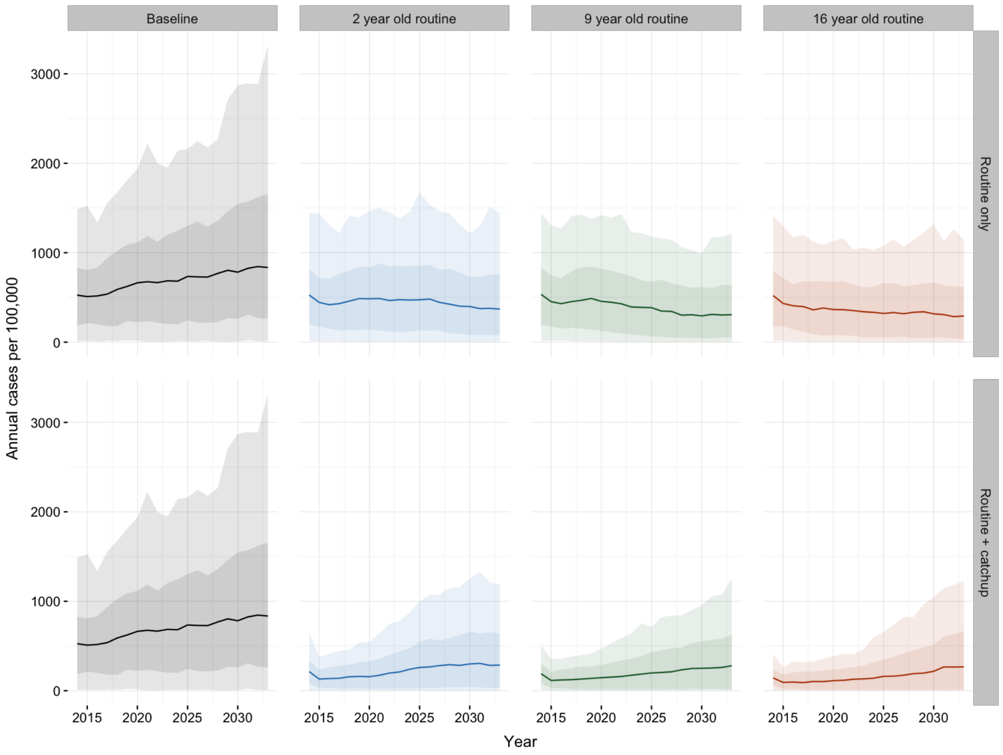

Figure H. Annual total cases per capita 95% (lightest) and 68% (darker) prediction intervals, and medians (solid line) for interventions with a durable vaccine. The panels are arranged by routine age of vaccination as well as non-intervention (rows) and routine versus routine-and-catchup campaigns (columns). Color is replicated from main text figures.

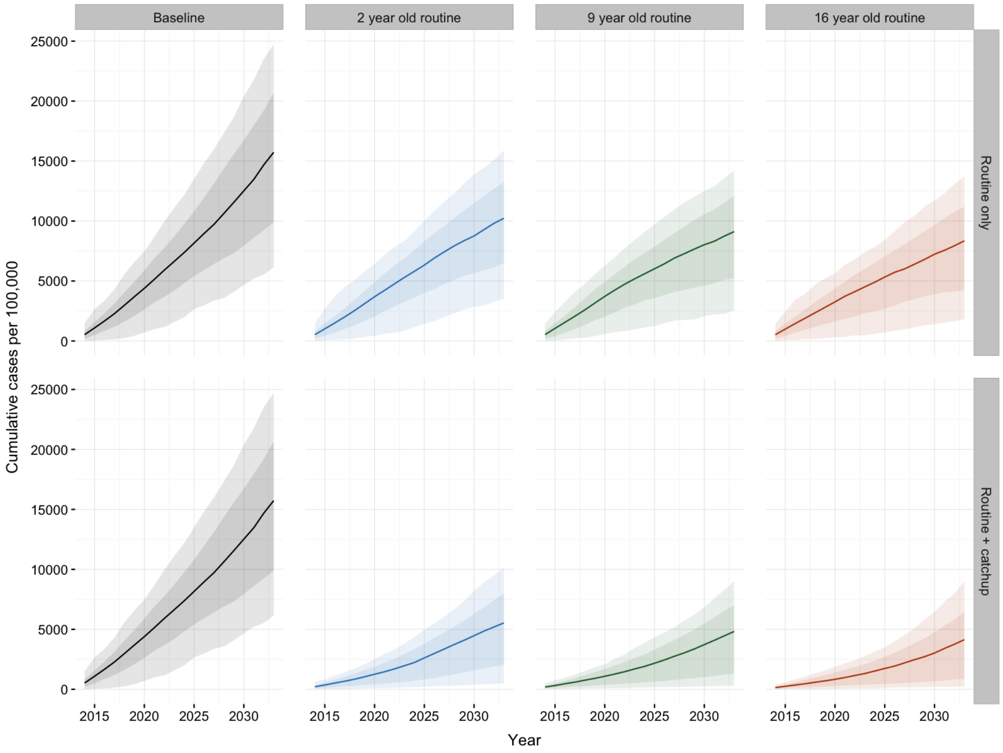

Figure I. Cumulative total cases per capita 95% (lightest) and 68% (darker) prediction intervals, and medians (solid line) for interventions with a durable vaccine. The panels are arranged by routine age of vaccination as well as non-intervention (rows) and routine versus routine-and-catchup campaigns (columns). Color is replicated from main text figures.

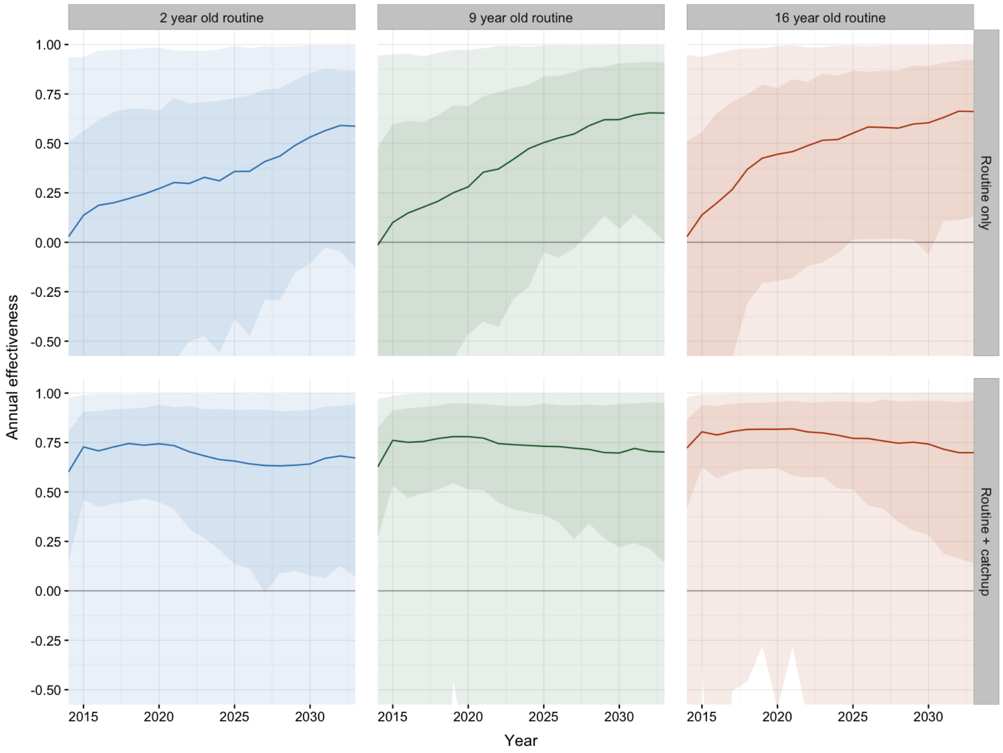

Figure J. Annual VE 95% (lightest) and 68% (darker) prediction intervals, and medians (solid line) for interventions with a durable vaccine. The panels are faceted by routine age of vaccination (rows) and routine versus routine and catchup campaigns (columns). Color is replicated from main text figures. Note that the lower forecast bound (2.5%) for the annual view is always negative, though it trends slowly upward (left out of frame), similar to the low bound (16%) for routine only strategies. For strategies with catchup campaigns, however, the resulting annual VE becomes less certain as the large initial coverage of seropositive vaccinees declines due to mortality.

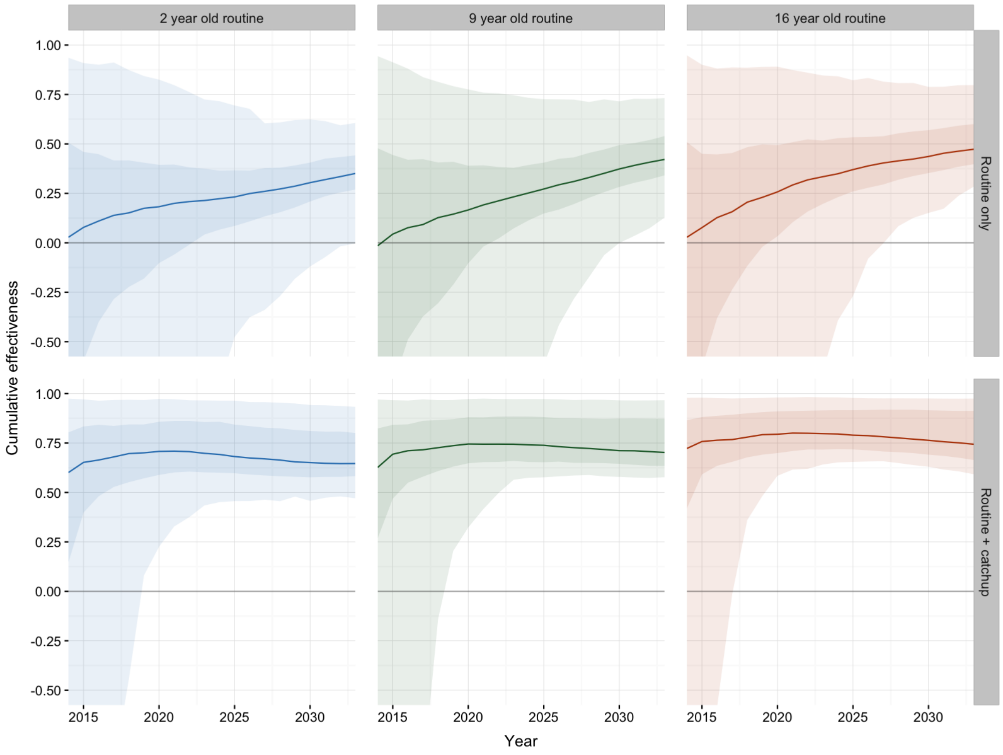

Figure K. Cumulative VE 95% (lightest) and 68% (darker) prediction intervals, and medians (solid line) for interventions with a durable vaccine. The panels are faceted by routine age of vaccination (rows) and routine versus routine and catchup campaigns (columns). Color is replicated from main text figures.

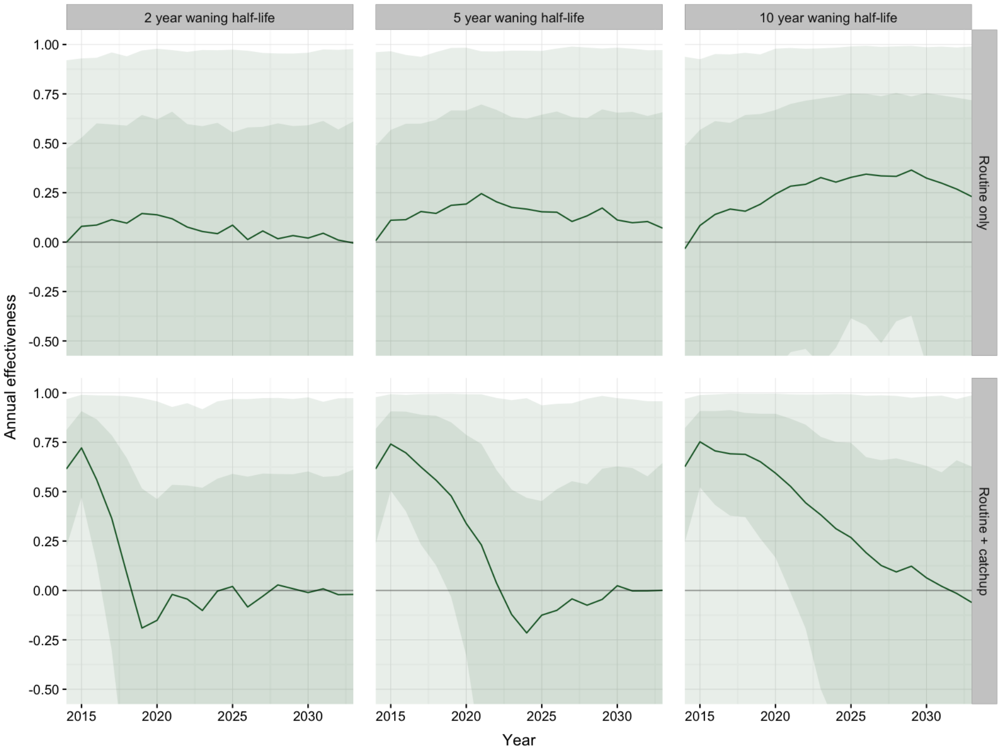

Figure L. Annual VE 95% (lightest) and 68% (darker) prediction intervals, and medians (solid line) for interventions with a waning vaccine and no booster vaccination campaigns. Routine age of vaccination is 9 years old. The panels are faceted by routine versus routine and catchup campaigns (rows) and waning half-life (columns). Color is replicated from main text figures. As noted in main text, a waning vaccine leads to limited VE, and when combined with a catchup campaign, leads to an expected temporary negative VE, which is not seen in any other scenario.

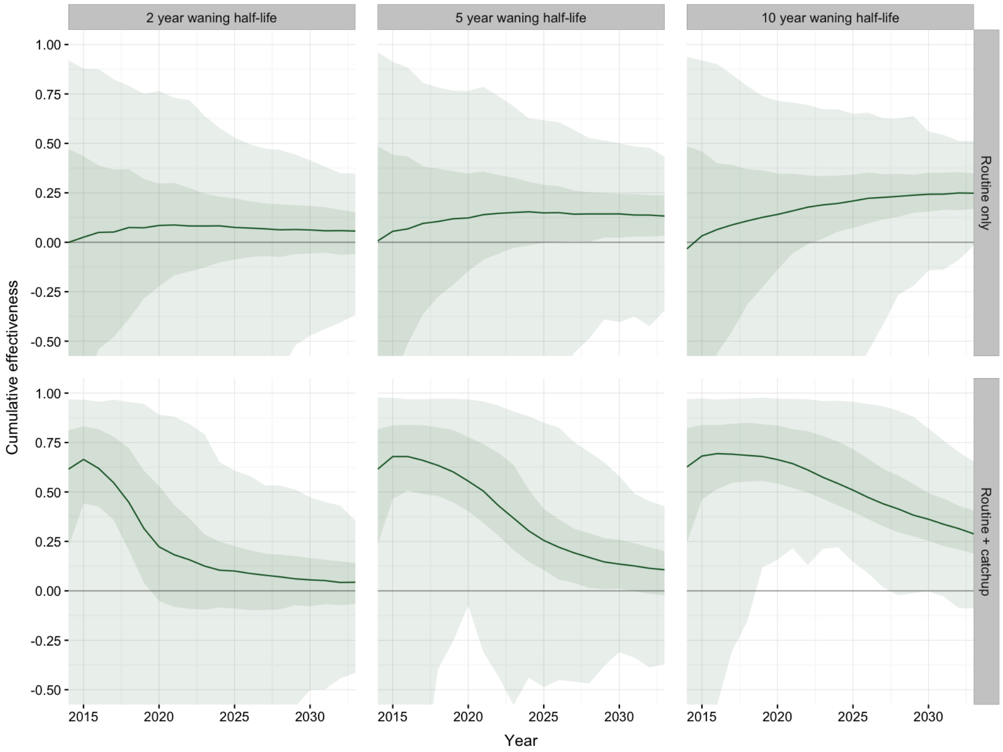

Figure M. Cumulative VE 95% (lightest) and 68% (darker) prediction intervals, and medians (solid line) for interventions with a waning vaccine and no booster vaccination campaigns. Routine age of vaccination is 9 years old. The panels are faceted by routine versus routine and catchup campaigns (rows) and waning half-life (columns). Color is replicated from main text figures. Note that even with the catchup campaigns crash in annual VE, all the still scenarios trend towards an expected small net benefit.

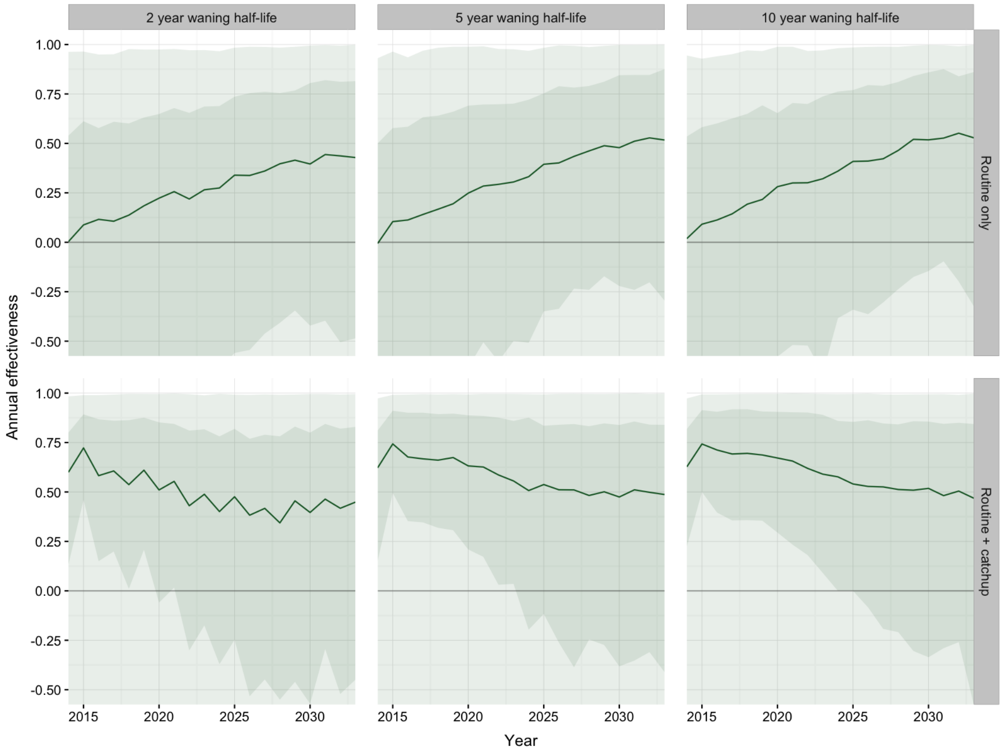

Figure N. Annual VE 95% (lightest) and 68% (darker) prediction intervals, and medians (solid line) for interventions with a waning vaccine with booster vaccination campaigns. Routine age of vaccination is 9 years old. The panels are faceted by routine versus routine and catchup campaigns (rows) and waning half-life (columns). Color is replicated from main text figures. The booster campaigns essentially restore performance to the level of a durable vaccine.

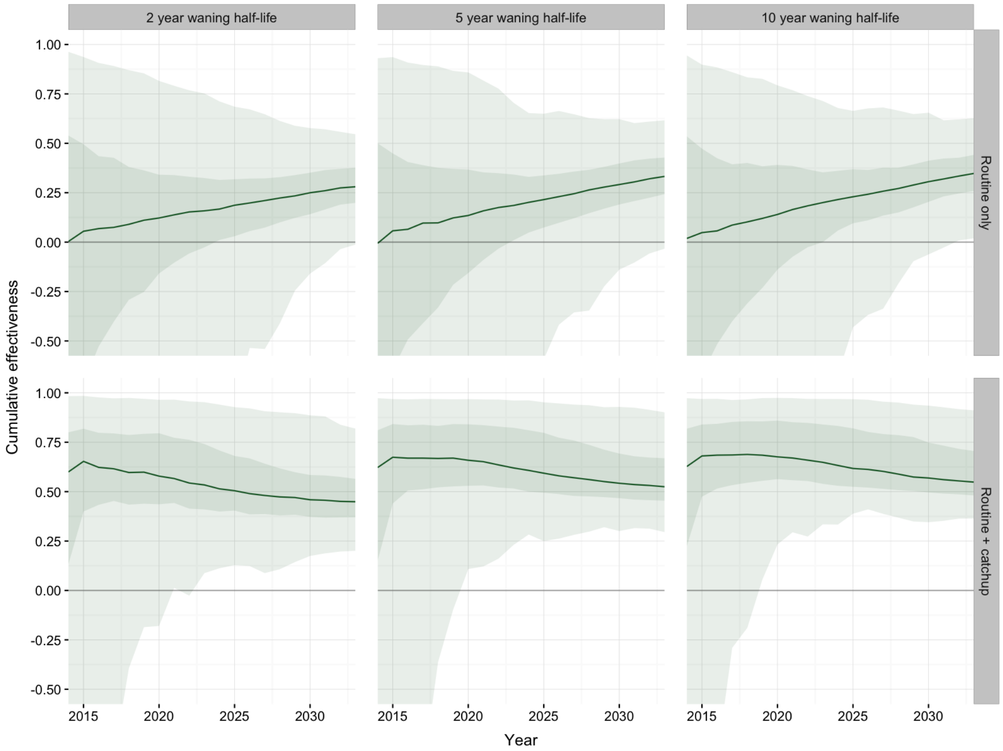

Figure O. Cumulative effectiveness 95% (lightest) and 68% (darker) prediction intervals, and medians (solid line) for interventions with a waning vaccine with booster vaccination campaigns. Routine age of vaccination is 9 years old. The panels are faceted by routine versus routine and catchup campaigns (rows) and waning half-life (columns). Color is replicated from main text figures.

S8 Fitting Period Detail

8242

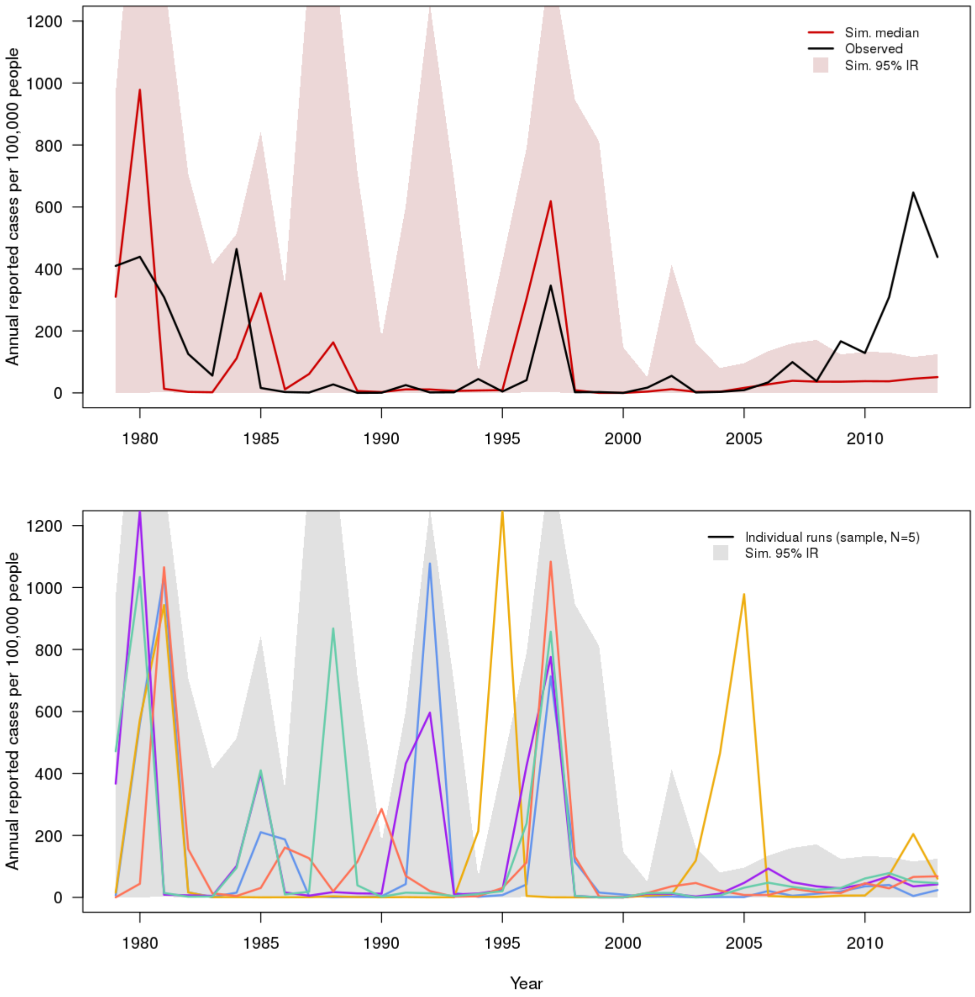

Figure P. Fitting period (detail). Annual observed and simulated median reported cases per capita during fitting period (top, reproduced from Fig. 3). A random sample of 5 simulated runs from different parameter combinations during the fitting period (bottom). Median epidemic sizes are a useful characterization of typical outcomes, but individual runs of a stochastic simulation can vary substantially from the median.

References

1. Chan M, Johansson MA. The Incubation Periods of Dengue Viruses. PLoS ONE. 2012;7(11).
2. Reich NG, Shrestha S, King AA, Rohani P, Lessler J, Kalayanarooj S, et al. Interactions between serotypes of dengue highlight epidemiological impact of cross-immunity. Journal of The Royal Society Interface. 2013;10(86):20130414.
3. Nisalak A, Endy TP, Nimmannitya S, Kalayanarooj S, Thisayakorn U, Scott RM, et al. Serotype-specific dengue virus circulation and dengue disease in Bangkok, Thailand from 1973 to 1999. The American Journal of Tropical Medicine and Hygiene. 2003;68(2):191–202. Available from: http://www.ajtmh.org/content/68/2/191.abstract.
4. Egger JR, Coleman PG. Age and clinical dengue illness. Emerging Infectious Diseases. 2007;13(6):924.
5. Chao DL, Halstead SB, Halloran ME, Longini IM Jr. Controlling Dengue with Vaccines in Thailand. PLoS Negl Trop Dis. 2012 10;6(10):e1876. Available from: http://dx.doi.org/10.1371%2Fjournal.pntd.0001876.
6. Dirección General de Epidemiología, SINAVE, Ministry of Health, Mexico. Dengue Morbidity Report; 2015.
7. Gómez-Dantés H. Dengue Seroprevalence study in urban settings in Yucatàn, Mexico. In preparation. 2015;.
8. Rojas DP, Pavia N, Feldstein L, Halloran ME, Longini IM, Gómez-Dantés H. Methodological approach for the evaluation of dengue control interventions: baseline field studies in Yucatan, Mexico. In preparation. 2015;.
9. Instituto Nacional de Estadística y Geographía. Banco de Información; 2015. http://www3.inegi.org.mx/sistemas/biinegi/.
10. Minnesota Population Center. Integrated Public Use Microdata Series, International: Version 6.3 [Machine-readable database]. University of Minnesota; 2014. https://international.ipums.org/international/.
11. Hijmans R. DIVA-GIS; 2013. Version 7.5. http://www.diva-gis.org/.
12. Earth Observation Group, NOAA National Geophysical Data Center. VIIRS DNB Cloud Free Composites; 2013. http://ngdc.noaa.gov/eog/viirs/download_monthly.html.
13. Directorio Estadístico Nacional de Unidades Económicas (DENUE); 2013. http://www3.inegi.org.mx/sistemas/mapa/denue/default.aspx.
14. Secretaría de Educación de Yucatán (SEGEY); 2013. http://www.educacion.yucatan.gob.mx/.
15. World Bank. Pupil-teacher ratio, primary; 2015. http://data.worldbank.org/indicator/SE.PRM.ENRL.TC.ZS.
16. Yasuno M, Tonn RJ. A study of biting habits of *Aedes aegypti* in Bangkok, Thailand. Bulletin of the World Health Organization. 1970;43(2):319.

17. Rueda L, Patel K, Axtell R, Stinner R. Temperature-dependent development and survival rates of *Culex quinquefasciatus* and *Aedes aegypti* (Diptera: Culicidae). Journal of Medical Entomology. 1990;27(5):892–898.

18. Climate Data Online, NOAA National Climatic Data Center. Daily summaries for Merida, MX; 2014. http://www.ncdc.noaa.gov/cdo-web/.

19. Shewchuk JR. Triangle: Engineering a 2D quality mesh generator and Delaunay triangulator. In: Applied Computational Geometry Towards Geometric Engineering. Springer; 1996. p. 203–222.

20. Harrington LC, Scott TW, Lerdthusnee K, Coleman RC, Costero A, Clark GG, et al. Dispersal of the dengue vector *Aedes aegypti* within and between rural communities. The American Journal of Tropical Medicine and Hygiene. 2005;72(2):209–220.

21. Styer LM, Carey JR, Wang JL, Scott TW. Mosquitoes do senesce: departure from the paradigm of constant mortality. The American Journal of Tropical Medicine and Hygiene. 2007;76(1):111–117.

22. Capeding MR, Tran NH, Hadinegoro SRS, Ismail HIHM, Chotpitayasunondh T, Chua MN, et al. Clinical efficacy and safety of a novel tetravalent dengue vaccine in healthy children in Asia: a phase 3, randomised, observer-masked, placebo-controlled trial. Lancet. 2015 06/11;384(9951):1358–1365. Available from: http://dx.doi.org/10.1016/S0140-6736(14)61060-6.

23. Villar L, Dayan GH, Arredondo-García JL, Rivera DM, Cunha R, Deseda C, et al. Efficacy of a Tetravalent Dengue Vaccine in Children in Latin America. New England Journal of Medicine. 2015;372(2):113–123. PMID: 25365753. Available from: http://dx.doi.org/10.1056/NEJMoa1411037.

24. Nishiura H, Halstead SB. Natural History of Dengue Virus DENV–1 and DENV–4 Infections: Reanalysis of Classic Studies. Journal of Infectious Diseases. 2007;195(7):1007–1013.

25. Montoya M, Gresh L, Mercado JC, Williams KL, Vargas MJ, Gutierrez G, et al. Symptomatic versus inapparent outcome in repeat dengue virus infections is influenced by the time interval between infections and study year. PLoS Negl Trop Dis. 2013;7(8):e2357.

26. Beaumont MA. Approximate Bayesian Computation in Evolution and Ecology. Annual Review of Ecology, Evolution, and Systematics. 2010;41(1):379–406. Available from: http://www.annualreviews.org/doi/abs/10.1146/annurev-ecolsys-102209-144621.

27. Csillery K, Blum MG, Gaggiotti OE, Francois O. Approximate Bayesian Computation (ABC) in practice. Trends Ecol Evol (Amst). 2010 Jul;25(7):410–418.

28. Geladi P, Kowalski BR. Partial least-squares regression: a tutorial. Analytica Chimica Acta. 1986;185:1 – 17. Available from: http://www.sciencedirect.com/science/article/pii/0003267086800289.

29. Farfán-Ale J, Loroño-Pino M. The incidence of dengue virus infection in children 8 to 14 years old residing in the urban and rural areas of the city of Merida, Yucatan. Boletin Medico del Hospital Infantil de Mexico. 1991;48(11):780–784.

30. Pachauri RK, Allen M, Barros V, Broome J, Cramer W, Christ R, et al.. Climate Change 2014: Synthesis Report. Contribution of Working Groups I, II and III to the Fifth Assessment Report of the Intergovernmental Panel on Climate Change. IPCC; 2014.

31. Mordecai EA, Paaijmans KP, Johnson LR, Balzer C, Ben-Horin T, Moor E, et al. Optimal temperature for malaria transmission is dramatically lower than previously predicted. Ecology letters. 2013;16(1):22–30.

32. Delatte H, Gimonneau G, Triboire A, Fontenille D. Influence of temperature on immature development, survival, longevity, fecundity, and gonotrophic cycles of *Aedes albopictus*, vector of chikungunya and dengue in the Indian Ocean. Journal of medical entomology. 2009;46(1):33–41.

33. Kekesi A, Shirah G, Radcliff MR, Potter G, Carriere L, Alder J, et al.. CMIP5: 21st Century Precipitation Scenarios. NASA's Goddard Space Flight Center Scientific Visualization Studio; 2016. http://svs.gsfc.nasa.gov/goto?4106.
